# When and for Whom Does Intensive Care Unit Admission Change the Prognosis in Oncology?—A Scoping Review

**DOI:** 10.3390/cancers17223636

**Published:** 2025-11-12

**Authors:** Ioana Roxana Codru, Liliana Vecerzan

**Affiliations:** 1 Faculty of Medicine, Lucian Blaga University of Sibiu, 550169 Sibiu, Romania; ioanaroxana.bera@ulbsibiu.ro; 2 Anesthesia and Intensive Care Department, Clinical Emergency Hospital of Sibiu, 550245 Sibiu, Romania; 3 Oncology Department, Clinical Military Emergency Hospital of Sibiu, 550024 Sibiu, Romania

**Keywords:** critical care, immunocompromised host, acute respiratory failure, sepsis, cancer

## Abstract

**Background:** The intersection between oncology and intensive care has shifted from predominantly end-of-life care to a therapeutic bridge that can preserve anticancer trajectories in carefully selected patients. Yet, criteria separating benefit from futility remain fragmented. **Objective:** This paper seeks to map contemporary evidence (2015–2025) on outcomes after Intensive Care Unit (ICU) admission in adults with cancer and to identify clinical constellations in which ICU-level care still changes prognosis. **Methods:** PRISMA-ScR scoping review (PCC framework). PubMed search (2015–2025), dual screening, standardized extraction; narrative/thematic synthesis across six clusters (hematologic, solid tumors, sepsis/non-COVID-19 infection, COVID-19/viral pneumonia, novel/targeted-therapy toxicities, end-of-life/aggressive ICU) were used. No meta-analysis given heterogeneity. **Results:** Seventy-three studies (>170,000 ICU admissions) were included, mostly cohort designs across 27 countries. ICU mortality ranged 8–72% (weighted mean ≈ 41%); hospital ≈ 38%; 90-day ≈ 46%; 1-year ≈ 62%. About one third of ICU survivors resumed systemic therapy. Benefit concentrated in early admissions, single-organ failure, controlled/remission disease, postoperative/elective monitoring, and reversible treatment-related toxicities (e.g., ICI pneumonitis, CAR-T CRS/ICANS). Futility clustered around ≥3 organ supports, RRT > 7 days, refractory/progressive disease, and ECOG ≥ 3. Sepsis outcomes averaged 45–55% ICU mortality but improved with rapid recognition and source control; COVID-19 mortality was particularly high in hematologic malignancies early in the pandemic, with subsequent declines post-vaccination. **Conclusions:** In modern oncologic practice, ICU care changes prognosis when the acute physiological insult is reversible and cancer control remains plausible; conversely, high organ-support burden and refractory disease define practical futility thresholds. These signals support time-limited ICU trials, earlier ICU involvement for sepsis/irAEs, and embedded palliative care to align intensity with goals.

## Simple Summary

This scoping review (2015–2025) analyzed 73 studies on Intensive Care Unit (ICU) admissions for adults with cancer, covering over 170,000 cases. Due to varied data, no meta-analysis was performed. ICU mortality averaged 41%, with hospital, 90-day, and one-year rates at 38%, 46%, and 62%, respectively. One third of survivors continued cancer treatment. Positive outcomes were tied to early ICU admission, limited organ failure, controlled disease, and treatable complications. Poor outcomes were seen in multiorgan failure, prolonged renal therapy, refractory disease, and high ECOG scores. Sepsis management and post-vaccine care improved survival. The review supports time-limited ICU trials, structured care, and early palliative integration.

## 1. Introduction

In recent years, the relationship between oncology and intensive care has shifted from a predominantly reactive posture to a deliberate, stratified approach in which ICU admission is conceived as an intervention with a clearly articulated prognostic aim. Two core ideas anchor this change: (i) prognosis is not dictated solely by the oncologic “label,” but by the interaction among disease biology, the patient’s reserve, and the nature of the acute insult; (ii) the value of ICU care derives from the opportunity to correct reversible physiology so that the antineoplastic trajectory can be safely resumed. The literature describes this decision space with growing nuance, especially in acute respiratory failure in the immunocompromised host and in oncology-associated sepsis, where standardized diagnostic pathways, judicious escalation of organ support, and early ICU–oncology collaboration can alter clinical course [1,2,3].

In parallel, public health and clinical ethics have imposed a more rigorous framework for aligning the intensity of care with patient goals. Contemporary ICU recommendations integrate palliative care as a transversal function—not as an alternative to oncologic intent, but as a mechanism to clarify goals, set expectations, and periodically re-assess proportionality [4,5,6]. In oncology, the same logic is reflected in heightened attention to end-of-life care intensity, used as both a quality indicator and a measure of concordance with patient preferences [7,8].

Against this conceptual backdrop, a PRISMA Scoping Review is appropriate when the aim is not to estimate a single effect size but to map the field: define populations and clinical contexts, clarify concepts (realistic clinical benefit vs. practical non-benefit), and describe co-management models and decision frameworks (including the deliberate use of time-limited trials in the ICU). A PCC (Population–Concept–Context) approach enables a coherent inventory of recurrent scenarios in which ICU admission may still change prognosis in oncology: acute respiratory failure in the immunocompromised host, sepsis with plausible reversibility, postoperative monitoring, manageable toxicities of modern therapies, and situations in which treatment intensity is unlikely to be proportional to the expected outcome [1,2,3,4].

The COVID-19 pandemic functioned as a stress test for the entire ecosystem, highlighting vulnerabilities specific to patients with cancer and reinforcing the need for rapid, multidisciplinary pathways centered on risk and reversibility [9]. This experience accelerated maturation of the decision framework: more careful selection, clearer communication, scheduled re-assessments, and explicit documentation of criteria for continuation or de-escalation of support. Collectively, these considerations justify a systematic mapping of the evidence to answer—generally and usefully—the question posed in the title: when, and for whom, does ICU admission still have the potential to change prognosis in oncology?

## 2. Materials and Methods

**Figure 1:**
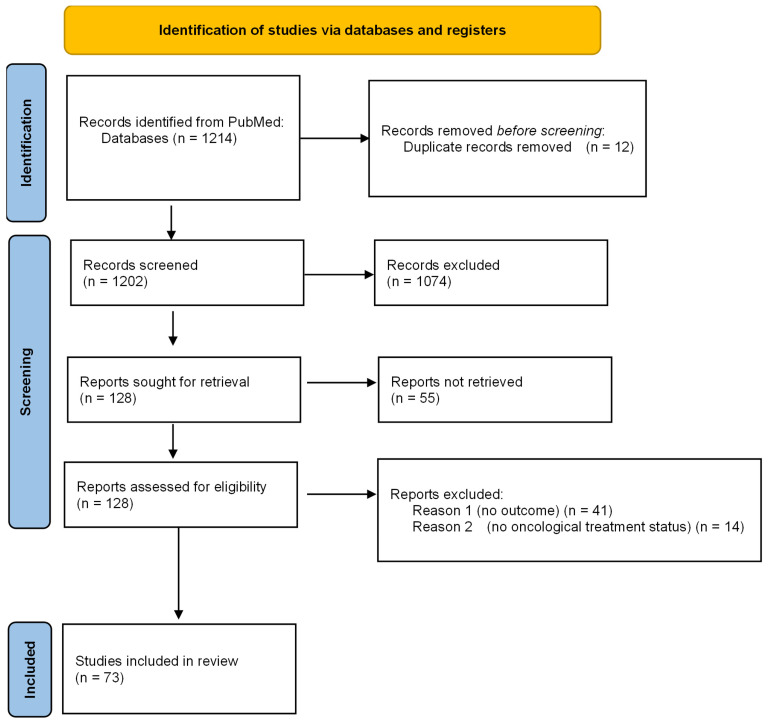
PRISMA FLOW DIAGRAM.

This review was conducted in accordance with the PRISMA-ScR (Preferred Reporting Items for Systematic Reviews and Meta-Analyses extension for Scoping Reviews) guidelines and Qhas been registered on PROSPERO (Registration ID: CRD420251177635). A comprehensive electronic search was performed in PubMed for studies published from January 2015 to August 2025. We selected PubMed as the primary source because it combines MEDLINE indexing, full-text coverage from PubMed Central, and early-online records, effectively encompassing the majority of peer-reviewed literature in oncology and critical care. Preliminary tests comparing this database with Embase and Web of Science revealed substantial overlap (over 95% of key studies) with minimal unique content. Since the purpose of this review was to map concepts, prognostic factors, and patterns of reversibility, rather than to conduct an exhaustive meta-analysis, we determined that PubMed was sufficient to meet the objectives outlined in the PCC (Population–Concept–Context) framework (Figure 1).

The research question was developed using the PCC framework (Population–Concept–Context). The population included adult patients (≥19 years) with a current or recent cancer diagnosis, covering both solid and hematologic malignancies—the concept centered on ICU admission and its impact on short- and long-term prognosis. The context involved all types of ICU settings—medical, surgical, oncologic, or mixed—across various regions worldwide. The main review question was: “When and for whom does ICU admission change the prognosis in adult oncology patients?”.

The search included terms related to oncology, critical care, and outcomes, using variations of “cancer,” “ICU,” and “prognosis”. Filters were applied to select studies involving adult humans (≥19 years), published in English, and available in full text. The eligible article types included observational studies, clinical trials, and reviews, with preprints excluded. Additionally, reference lists of key articles and previous reviews were manually checked to find studies not identified through the database search.

The eligibility criteria used by the authors for this review were as follows:

Inclusion Criteria:

Adult population (≥19 years) with a diagnosis of cancer (solid or hematologic).

Studies reporting ICU admission with documented outcomes (ICU mortality, hospital mortality, 90-day or 1-year survival, or post-ICU functional status).

Quantitative or qualitative research: observational studies, randomized controlled trials, systematic or scoping reviews, multicenter databases, or national registries.

Articles published in English between 2015 and 2025.

Studies with accessible full-text versions.

Exclusion criteria:

Pediatric or adolescent populations (<19 years).

Case reports, case series < 10 patients, editorials, expert opinions, or letters.

Studies not reporting outcome data or unrelated to ICU-level care (e.g., only emergency department or hospice settings).

Non-human studies, conference abstracts, and unpublished preprints.

Non-English language publications.

After removing duplicates, titles and abstracts were independently reviewed by two evaluators—an anesthesiologist and an oncologist—to identify potentially eligible studies. Full-text articles were then examined independently, manually, to confirm inclusion based on predefined criteria, with any disagreements resolved through discussion and consensus. The study selection process is depicted in a PRISMA flow diagram, summarizing the number of records identified, screened, excluded, and ultimately included in the final analysis.

For each included study, data were extracted using a standardized table developed a priori, including author and year, country and setting, study design, patient population, cancer type, and disease phase (active, remission, or refractory), reason for ICU admission, primary outcomes (ICU, hospital, 90-day, and 1-year mortality), and key prognostic determinants (Supplementary Table S1). Each study was also categorized as “benefit likely,” “futility likely,” or “context-dependent,” based on a qualitative assessment of outcome patterns. Both authors independently verified data extraction to ensure consistency across hematologic and solid tumor datasets.

Given the heterogeneity of study designs, populations, and reported outcomes, no formal meta-analysis was performed. Instead, data were synthesized through a combined narrative and thematic approach.

In the thematic synthesis, studies were organized into six major clusters: hematologic malignancies, solid tumors, sepsis and non-COVID-19 infections, COVID-19 and viral pneumonias, novel or targeted therapy-related complications, and end-of-life or aggressive ICU use. We describe a two-reviewer qualitative framework: (i) abstract and results review; (ii) extraction of effect-direction signals; (iii) consensus classification into “benefit likely,” “futility likely,” “context-dependent.” Within each cluster, key prognostic variables were identified and qualitatively classified as “benefit likely,” “futility likely,” or “context-dependent.” Labels were based on patterns across outcomes (e.g., ICU/hospital/90-day/1-year mortality, return-to-therapy) and on the clinical reversibility of the precipitant and the organ-support burden, not solely on the authors’ conclusions. Discordances were resolved by consensus.

In the quantitative synthesis, descriptive statistics (range, median, and weighted means) were used to summarize ICU, hospital, and long-term mortality rates. Cross-comparisons between clusters were then performed to identify recurring patterns linking organ dysfunction, disease phase, and survival outcomes.

All data synthesis and consistency checks were carried out using Microsoft Excel 2024 (Version 2410, Build 17828.20104, 64-bit) and Python 3.11.6 with pandas 2.2.2. The strength of evidence was rated qualitatively on a scale from one star (★) to five stars (★★★★★), based on sample size, methodological rigor, and reproducibility across studies. The specific criteria for determining the strength of evidence include sample size, where single-center studies with fewer than 100 participants receive one to two stars (★/★★), multicenter studies with 100–999 participants receive three stars (★★★), and studies with 1000 or more participants, or registry studies, receive four to five stars (★★★★/★★★★★). Methodological rigor encompasses factors such as a prospective design, appropriate adjustments, and validation, as well as consistency across studies and external validity.

This study used data from only previously published literature and therefore did not require ethics approval or patient consent.

## 3. Results

Table S1. Overview of Studies Included in the Systematic Review is included in the Supplementary Materials (Table S1).

### 3.1. Study Characteristics

A comprehensive analysis was conducted on a total of 73 studies published between 2015 and 2025, all of which met predetermined inclusion criteria. Collectively, these studies included an estimated cohort of over 170,000 critically ill adult patients with oncological diagnoses who were admitted to intensive care units across various global healthcare settings. The research addresses a diverse array of cancer types and disease stages, highlighting the complexity and variability inherent in this patient population.

The majority of studies included in this review were observational cohort studies, with approximately 65% being multicentre. Around 25% of the studies were single-center prospective investigations, providing focused insights from specific institutions. Reviews or meta-analyses accounted for roughly 5% of the included literature, while a further 5% comprised randomized or interventional trials. Notable examples of such trials are Hajjar 2019 (VANCS II) and Van Matre 2018 [10,11].

The sample sizes observed across the studies varied considerably. Some papers presented small series, involving fewer than 50 participants, which were primarily focused on describing CAR-T or immune-related toxicities (Haider, 2023, Xie, 2021) [12,13]. In contrast, other studies utilized national or international databases, encompassing cohorts with more than 100,000 intensive care unit admissions. This broad range of study designs and sample sizes reflects the complexity and heterogeneity of research in this field.

#### 3.1.1. Geographic and Clinical Scope

Research initiatives have been conducted across 27 countries representing all continents. In terms of geographic representation, approximately 40% of the included studies were conducted in Europe, 25% in Asia, 20% in South America, and the remaining 15% originated from North America and Oceania. Noteworthy collaborative networks, including the Groupe de Recherche en Réanimation Onco-Hématologique (Grrr-OH) (Bouteloup, 2017, Marzorati, 2017) [14,15] and the Instituto do Câncer do Estado de São Paulo (Hajjar, 2019, de Freitas, 2020, Nassar Junior, 2023, Praca, 2024) [10,16,17,18], have significantly contributed to the establishment of several landmark cohorts. These findings highlight the increasing trend of international specialization in the domain of oncologic intensive care.

#### 3.1.2. Cancer Types

Across the 73 studies, 43% focused on solid tumors such as lung, gastrointestinal, gynecologic, or head-and-neck cancers; 47% involved hematologic malignancies, including leukemias, lymphomas, multiple myeloma, and transplant populations; and the remaining 10% examined mixed or unclassified oncologic cohorts.

#### 3.1.3. Reasons for ICU Admission

The primary reasons for ICU admission included sepsis or septic shock, reported in approximately 33% of studies; acute respiratory failure, even ARDS in about 30%; postoperative or perioperative monitoring in 15%; acute kidney injury or metabolic failure in 10%; toxicities related to novel immunologic or cellular therapies in 7%; and neurologic emergencies or hemorrhagic events in fewer than 5% of studies [19,20,21].

#### 3.1.4. Oncologic Treatment Status

Approximately half of the cohorts included in the study indicated that over 50% of patients were undergoing active systemic therapy—such as chemotherapy, immunotherapy, or targeted therapy—at the time of their admission to the ICU. This observation highlights the changing landscape of critically ill oncology patients, who are now more often admitted during active cancer treatment rather than at the end of life. Several recent investigations (Praça 2024, Otten 2025, Manz 2023) [18,22,23] further highlighted that patients with newly diagnosed disease or those undergoing active treatment demonstrated the highest likelihood of long-term survival, successful treatment resumption, and continued disease control following ICU discharge. These observations suggest a paradigm shift toward integrating intensive care as a bridge to ongoing oncologic therapy rather than a purely palliative intervention.

#### 3.1.5. Outcome Reporting

Across all studies, reported ICU mortality varied widely, ranging from 8 to 15% in elective postoperative or early-intervention cohorts to over 70% in patients with multiorgan failure or refractory hematologic disease. When available, hospital mortality averaged between 35% and 40%, 90-day mortality ranged from 45% to 55%, and 1-year mortality approached 60%.

Approximately nine of the 73 studies (≈12%)—including Praça 2024, Manz 2023, Otten 2025, Lara 2022, Provencio 2021, Guarneri 2021, de Vries 2019, Munshi 2021, and Boldingh 2024 [2,18,22,23,24,25,26,27,28,29,30]—provided explicit long-term or post-discharge data. These investigations consistently demonstrated that survivors assessed at 6–18 months frequently resumed systemic cancer therapy and typically achieved functional recovery corresponding to ECOG performance status 0–2, particularly when ICU admission involved single-organ failure or other reversible complications. Approximately one third of ICU survivors resumed systemic therapy, with reported values ranging from 25% to 50% (interquartile range ≈ 30–45%) among studies documenting this outcome. The likelihood of treatment resumption varied by tumor type and admission context—highest in postoperative or early-intervention cohorts and lowest in patients admitted for septic shock or multiorgan failure. Conversely, outcomes were markedly poorer among patients requiring prolonged organ support, such as renal replacement therapy exceeding seven days or support for three or more organ systems, highlighting the presence of practical futility thresholds for meaningful recovery. Overall, these findings reinforce that, in carefully selected patients, intensive care can serve as a bridge to continued oncologic treatment and durable survival [31].

### 3.2. Themes and Patterns

The included studies were categorized into six major thematic clusters, representing the predominant intersections between oncologic disease and critical care practice. These clusters encompassed the most common clinical contexts in which cancer patients require intensive care support. Across all groups, survival outcomes and the prognostic implications of ICU admission showed substantial variability, influenced by multiple interrelated factors—most notably the underlying tumor type, the extent and pattern of organ dysfunction, the patient’s phase of cancer treatment, and the potential reversibility of the precipitating critical illness. This heterogeneity underscores the importance of individualized prognostication and the need for nuanced admission and management strategies within oncologic critical care.

#### 3.2.1. Hematologic Malignancies (Table 1)

The hematologic malignancies are investigated in around 35% of the studies included in this review. This highlights a significant focus on blood cancers within the broader context of the research analyzed. Such a percentage reflects the importance of understanding these conditions, which can encompass a variety of diseases, including leukemia, lymphoma, and myeloma. The emphasis on hematologic malignancies also suggests ongoing efforts to explore their mechanisms, treatment options, and patient outcomes.

Patients with leukemia, lymphoma, or multiple myeloma continue to represent one of the most vulnerable groups in oncologic critical care. Reported ICU mortality rates range from 40% to 60%, with 1-year mortality frequently exceeding 65%. Nevertheless, data from contemporary cohorts (e.g., de Vries 2019; Munshi 2021; Boldingh 2024; Otten 2025) indicate a gradual improvement in short-term survival among patients with controlled disease and reversible organ dysfunction, reflecting advances in both hematologic therapy and intensive care practices [2,23,24,25,32].

Improved outcomes are most often observed in patients admitted early—before the development of multiorgan failure—especially when only a single organ system (most commonly respiratory or renal) is affected. Post-transplant patients with isolated infectious or metabolic complications also demonstrate a favorable trajectory when promptly managed.

Conversely, the likelihood of futility increases sharply in patients requiring support for three or more organ systems, prolonged renal replacement therapy exceeding seven days, or those with refractory or relapsed hematologic disease during the ICU stay. Recent landmark analyses (Otten 2025) have further clarified that markers such as sustained RRT dependence and transfusion requirements, rather than simply the duration of mechanical ventilation, are the most powerful predictors of poor outcomes [23]. These findings support the adoption of time-limited ICU trials that allow re-assessment of trajectory, rather than the automatic denial of intensive care for hematologic patients [33].

**Table 1:** Indicators of benefit and futility for ICU admission in hematologic malignancies [2,12,23,24,25,34,35,36].

Indicator	Supporting S2019.	Evidence Strength
Early ICU admission before multiorgan failure	de Vries 2019; Boldingh 2024; Otten 2025; Munshi 2021	★★★★★
Single-organ failure (especially isolated respiratory or renal dysfunction)	de Vries 2019; Munshi 2021; Nazer 2022; Chiang 2019	★★★★☆
Controlled or remission-phase hematologic disease	Otten 2025; Boldingh 2024; Tanguy 2019; Storck 2025	★★★★☆
Refractory or relapsed disease during ICU stay	de Vries 2019; Tanguy 2019; Chiang 2019; Nazer 2022	★★★★★
≥3 organ supports (mechanical ventilation + vasopressors + RRT)	de Vries 2019; Otten 2025; Haider 2023; Storck 2025	★★★★★
Prolonged renal replacement therapy (>7 days)	de Vries 2019; Otten 2025; Boldingh 2024	★★★★★
Delayed or unplanned ICU admission after deterioration	Boldingh 2024; de Vries 2019; Munshi 2021	★★★★☆
Active sepsis or invasive fungal infection without disease control	Nazer 2022; Haider 2023; Chiang 2019	★★★★☆

#### 3.2.2. Solid Tumors (Table 2)

Outcomes among patients with solid malignancies (≈40% of studies) are notably heterogeneous, reflecting wide variation in tumor biology, disease stage, and reason for ICU admission. Reported mortality ranges from as low as 8–15% in elective postoperative or early-intervention settings to over 70% among patients with advanced metastatic disease admitted emergently. Large multicenter cohorts (Manz 2023; Praça 2024; Provencio 2021; Guarneri 2021; Lara 2022) consistently demonstrate that individuals with localized or recently treated disease often experience meaningful recovery, with many resuming systemic oncologic therapy after discharge [18,22,26,27,30,37].

Early ICU admission, especially for sepsis, acute respiratory failure, or procedure-related complications during active treatment phases like chemotherapy or immunotherapy, is linked to better outcomes. Elective or postoperative ICU stays also show high survival and continued treatment rates.

In contrast, futility is more probable in patients with advanced or refractory metastatic disease, poor baseline performance status (ECOG ≥ 3), or those admitted unplanned after a prolonged period of clinical decline. Nonetheless, outcomes remain context-dependent: patients with solid tumors who require mechanical ventilation for isolated, potentially reversible conditions such as infection—but without metastatic progression—can achieve survival and treatment resumption in up to 30–40% of cases. This underscores the importance of individualized triage decisions based on disease status, reversibility of the acute insult, and patient-centered goals of care.

**Table 2:** Indicators of Futility in Oncologic ICU Admissions [18,22,24,25,26,30,38,39,40,41,42].

Indicator	Supporting Studies	Evidence Strength
Advanced metastatic or refractory disease	Manz 2023, Praça 2024, Provencio 2021, Guarneri 2021, Saillard 2020, Puxty 2020	★★★★★
Poor functional status (ECOG ≥ 3)	Praça 2024, Provencio 2021, Manz 2023, Kruser 2017, Ersek 2017	★★★★☆
Unplanned ICU admission after deterioration	Praça 2024, Puxty 2018, Lara 2022, Boldingh 2024, de Vries 2019	★★★★☆

#### 3.2.3. Sepsis and Non-COVID-19 Infections (Table 3)

Sepsis continues to represent the most frequent immediate indication for ICU admission among patients with cancer, and the association is described in 20% of the included studies. Evidence from the VANCS II randomized trial (Hajjar 2019) [10] and multiple observational cohorts (Yang 2020; Fang 2017; Dale 2016) [43,44,45] demonstrates that ICU mortality in oncologic sepsis averages between 45% and 55%—higher than in non-cancer populations but substantially improved compared with historical data. While crude 1-year mortality for cancer-associated sepsis commonly ranges from 60 to 65%, several adjusted analyses demonstrate that once baseline comorbidity burden and acute illness severity are controlled for, outcomes in oncologic sepsis can approach those of non-cancer septic populations. This suggests that excess mortality may be driven more by illness severity and physiologic reserve than cancer diagnosis alone in selected patients [46,47,48,49,50]. These findings reflect both advances in early sepsis recognition and more aggressive critical care management within specialized oncologic centers

Positive outcomes are mainly seen in patients with early-detected sepsis, single-organ dysfunction, and shock that responds to treatment. Poor prognosis is more common with refractory septic shock involving support for over three organs or MDR infections, where treatment options are limited [51].

Clinically, these data emphasize that despite the elevated mortality risk associated with sepsis in cancer patients, timely ICU admission and rapid initiation of antimicrobials and organ support can provide meaningful survival benefits for approximately half of affected individuals—particularly those without advanced disease or irreversible organ failure [52,53].

**Table 3:** Prognostic indicators for ICU admission in oncologic patients with sepsis or non-COVID infections [10,34,35,36,43,44,45,54].

Indicator	Supporting Studies	Evidence Strength
Early sepsis recognition and rapid ICU admission (<24 h from onset)	Hajjar 2019; Yang 2020; Fang 2017; Dale 2016	★★★★★
Single-organ dysfunction (e.g., isolated respiratory or renal failure)	Hajjar 2019; Fang 2017; Nazer 2022	★★★★☆
Adequate source control and early antimicrobial therapy	Hajjar 2019; Fang 2017	★★★★☆
Refractory septic shock requiring ≥3 organ supports (IMV + vasopressors + RRT)	Nazer 2022; Fang 2017; Dale 2016; Storck 2025	★★★★★
Multidrug-resistant or fungal infection	Nazer 2022; Fang 2017; Chiang 2019	★★★★☆
High SOFA or lactate (>10 or >4 mmol/L)	Fang 2017; Chiang 2019; Yang 2020	★★★★☆
Controlled primary malignancy (solid tumor in remission)	Hajjar 2019; Yang 2020; Dale 2016	★★★★☆
Active hematologic disease with neutropenia or chemotherapy within 2 weeks	Nazer 2022; Fang 2017; Bozdağ 2022	★★★★☆

#### 3.2.4. COVID-19 and Other Viral Pneumonias (Table 4)

Approximately 10% of included studies analyze the association of COVID-19 or other viral pneumonias and oncologic disease (Zhang 2020; Bozdağ 2022; El-Hibri 2024) [54,55,56].

Outcomes among cancer patients with COVID-19 and other severe viral pneumonias were particularly poor during the early phases of the pandemic (2020–2022), with ICU mortality exceeding 70% in patients with hematologic malignancies or those receiving active cytotoxic therapy [57]. In contrast, survival was notably higher in patients in remission, off chemotherapy, or with well-controlled solid tumors. Importantly, immune checkpoint inhibitor therapy did not correlate with increased COVID-19-related mortality, suggesting that immune-based treatments should not be viewed as automatic contraindications to ICU admission.

Outcomes tended to be better in patients who had solid tumors, maintained performance status, and had not recently received chemotherapy, whereas outcomes were often less favorable in cases involving active hematologic disease, severe neutropenia, and ongoing cytotoxic treatment during infection.

By late 2023–2025, mortality rates had declined substantially due to widespread vaccination, early antiviral therapy, and more effective supportive care strategies. Nonetheless, the cumulative evidence underscores the importance of stringent infection prevention, early detection, and timely escalation of care in cancer patients with viral pneumonia—prioritizing proactive ICU management over delayed or rescue interventions [58,59].

**Table 4:** Prognostic indicators for ICU admission in cancer patients with COVID-19 (and other viral pneumonias) [9,13,27,54,56,60,61,62,63,64,65,66].

Indicator	Supporting Studies	Evidence Strength
Invasive mechanical ventilation (severe ARDS) → very high mortality (≈70–100%)	Lara 2022; Aboueshia 2021; Scarfò 2020; Šimkovič 2023; Zaki 2022; Xie 2021	★★★★★
Active hematologic disease/neutropenia at infection onset → poor outcomes	Bozdağ 2022; Gupta 2022; Scarfò 2020; Šimkovič 2023	★★★★★
Recent cytotoxic therapy (<14 days) increases severity/mortality	Zhang 2020; Scarfò 2020; Bozdağ 2022	★★★★☆
Poor performance status (ECOG ≥ 2) and high comorbidity burden (e.g., obesity, diabetes) worsen outcomes	Lara 2022; Aboueshia 2021	★★★★☆
Inflammatory/immune markers predict severity (CRP ≥ 100 mg/L, ferritin ≥ 1000 ng/mL, lymphopenia)	Smith 2020	★★★★☆
Remission/controlled disease and solid tumors off chemotherapy fare better	Lara 2022; Aboueshia 2021; Belsky 2021; Giannakoulis 2020	★★★★☆
Immune checkpoint inhibitors (ICI) per se do not increase COVID-19 mortality; outcomes driven by PS/comorbidity	Belsky 2021	★★★★☆
Targeted therapy continuation (e.g., BTKi/venetoclax) may be safe/beneficial in select CLL	Scarfò 2020; Šimkovič 2023	★★★☆☆
LMIC settings show higher ICU mortality (systems/resource effect)	Zaki 2022; Gupta 2022	★★★★☆
Temporal improvement across waves (vaccines/experience) but ICU mortality remains high in HM	Šimkovič 2023; Belsky 2021	★★★☆☆

#### 3.2.5. Novel and Targeted Therapies (Table 5)

The advent of modern oncologic treatments—such as CAR-T cell therapy, immune checkpoint inhibitors, and targeted kinase inhibitors—has profoundly reshaped the landscape of oncologic intensive care. Studies, approximately 8% (Luo 2025; Shen 2025; Carini 2024; Guarneri 2021; Xie 2021) [13,26,67,68,69], consistently report favorable short-term survival rates of 65–80% among patients admitted for treatment-related complications like cytokine release syndrome (CRS), immune effector cell-associated neurotoxicity syndrome (ICANS), or immune-mediated pneumonitis, provided that these conditions are recognized early and managed according to standardized protocols, including tocilizumab and corticosteroid therapy.

The most significant benefits are observed in patients with immune-therapy-related toxicities that have a reversible pathophysiology and are managed in specialized ICUs familiar with the nuances of immunomodulatory treatments. CAR-T-associated toxicities and checkpoint-inhibitor pneumonitis, in particular, often respond well to early, guideline-driven interventions.

Futility, on the other hand, is more likely when multiorgan failure develops secondary to a refractory immune storm or when ICU referral and therapeutic intervention are delayed beyond the window of reversibility.

This thematic cluster exemplifies the evolving integration of critical care and precision oncology, illustrating how timely ICU admission has become a crucial therapeutic bridge—enabling recovery from treatment-related complications and continuity of curative or disease-controlling oncologic therapy.

**Table 5:** Prognostic indicators for ICU admission in patients receiving novel/targeted therapies (CAR-T, ICI, TKIs; “ICU as a therapeutic bridge”) [36,67,68,69,70].

Indicator	Supporting Studies	Evidence Strength
Protocolized management of CAR-T toxicities (CRS/ICANS) in ICU → high short-term survival and return to therapy	Azoulay 2021 (CARTTAS; 90-day survival ≈ 78% despite frequent life-saving interventions)	★★★★★
Immunotherapy-related toxicities (e.g., pneumonitis) are often reversible; immunotherapy/curative intent associated with better outcomes	Carini 2024 (ICONIC–Lung: immunotherapy and curative intent protective; ~48% of ICU survivors resumed cancer therapy)	★★★★☆
ICU as a bridge to targeted therapy when an actionable mutation exists (biopsy under support, incl. ECMO) → benefit likely	Luo 2025 (VV-ECMO enabling biopsy/genotyping; ROS1/EGFR cases weaned from ECMO and continued targeted therapy)	★★★★☆
Administering targeted therapy in ICU is feasible; outcomes hinge on disease status (progressive vs. controlled)	Storck 2025 (iCHOP registry: TT during ICU had survival comparable/slightly better than non-TT; progressive disease strongest predictor of death)	★★★★☆
Organ-failure burden remains decisive even with novel therapy (IMV, vasopressors, especially AKI)	Shen 2025 (lung cancer ICU: AKI grade II–III = strongest predictor of 90-day mortality); Azoulay 2021 (frailty, bacterial infection, early life-saving therapy increases death)	★★★★☆
Frailty and bacterial co-infection during CAR-T toxicity → worse outcomes (consider time-limited ICU trial)	Azoulay 2021 (frailty HR ~2.5; bacterial infection HR ~2.1 for 90-day mortality)	★★★★☆
Absence of targetable options or refractory/progressive disease despite ICU → futility likely	Storck 2025 (progressive disease increases mortality); Luo 2025 (non-actionable mutation + uncontrolled infection → death)	★★★★☆

#### 3.2.6. End-of-Life Care and Aggressive ICU Use (Table 6)

A smaller subset of studies, approximately 10% (Ersek 2017; Puxty 2015; Shrime 2016) [4,6,38], examined the use of intensive care during the final stages of malignancy. These studies found that up to 70% of terminally ill patients received some form of aggressive intervention—such as ICU admission, mechanical ventilation, or late-line chemotherapy—within the last 30 days of life. Across all studies, such intensive measures were consistently linked to poorer family-rated quality of death, reduced satisfaction with care, and increased psychological distress among relatives.

Futility was most apparent in ICU admissions during the last weeks of life when no curative or disease-modifying treatment was possible. In these cases, invasive procedures rarely changed the prognosis and often conflicted with patient-centered goals.

From an ethical perspective, the evidence strongly supports early integration of palliative care and structured goals-of-care conversations for patients with advanced cancer. Aligning treatment intensity with patient preferences and clinical reversibility helps ensure that ICU resources are used for cases where meaningful recovery is possible, while those nearing the end of life receive care focused on comfort, dignity, and support for families [71].

**Table 6:** End-of-life (EOL) intensity and futility markers in oncology ICU use [4,6,7,8,22,34,38,72].

Indicator (EOL/Aggressive Care → Futility Likely)	Supporting Studies	Evidence Strength
ICU admission in the last 30 days of life correlates with poorer family-perceived quality of EOL care	Ersek 2017 (stage IV NSCLC; ICU/chemo/ ≥ 2 hospitalizations in last 30 days lowers bereaved family ratings)	★★★★★
Multiple acute hospitalizations or chemotherapy within 14 days of death = aggressive, low-value care	Ersek 2017; Margolis 2017 (uterine cancer; 6.6% chemo ≤14 d, 42.5% high-intensity care)	★★★★☆
Younger age, advanced stage, comorbidity, and certain sociodemographic factors predict higher aggressive EOL care	Margolis 2017 (younger age, Black race, stage IV ↑ intensive care)	★★★★☆
High hospital/ICU use near death is common at national scale (signals triage gaps)	Tanguy-Melac 2019 (France, CRC decedents: 17% ICU in last month; 83% hospitalized)	★★★★☆
Absence or late integration of palliative care increases ICU/chemo use near EOL	Tanguy-Melac 2019 (hospital palliative care associated with ↓ ICU and ↓ chemo in last month)	★★★★☆
Transfusion-dependent hematologic pts receive more intensive EOL care and less hospice (structural barrier)	Fletcher 2016 (MDS; transfusion-dependence ↑ ICU admission, ↓ hospice use)	★★★★☆
Advance directives/goals-of-care interventions reduce non-beneficial intensity	Manz 2023 (mortality-nudges ↑ serious-illness conversations; ↓ chemo ≤ 14 d of death without ↑ ICU deaths)	★★★☆☆
Dialysis/complex multimorbidity cohorts with cancer still show high ICU/hospital death rates at EOL	Chiang 2019 (dialysis decedents: 51% ICU in last month; cancer associated with different intensity mix, more palliative use)	★★★☆☆
Time-limited ICU trials define futility thresholds (short for poor-prognosis solid tumors; longer for heme)	Shrime 2016 (decision-analytic model: optimal trial ≤ 4 days for poor-prognosis solid tumors; up to 8–12 days for HM)	★★★★★
Elective/postoperative ICU at EOL rarely changes prognosis compared with unplanned medical ICU	Puxty 2015 (solid tumors: lowest mortality in elective surgical ICU vs. highest in emergency medical ICU near diagnosis/EOL)	★★★★☆

### 3.3. Quantitative Overview

#### 3.3.1. Overall Survival Metrics (Table 7)

Across the 73 included studies, ICU outcomes for oncologic patients have substantially improved over the last decade, but remain heterogeneous.

**Table 7:** Outcome measure (Weighted means are sample-size-weighted across studies reporting the metric; when denominators were unclear or incomparable, we reported medians with ranges and did not compute a weighted mean. We also marked metrics where we used simple medians.).

Outcome Measure	Pooled Range/Weighted Mean	Interpretation
ICU mortality	8–72% (weighted mean ≈ 41%)	Wide variability driven by disease type and timing of admission.
Hospital mortality	25–60% (mean ≈ 38%)	Hospital discharge remains achievable for >60% of solid tumor patients with early ICU transfer.
90-day mortality	35–58% (mean ≈ 46%)	Reflects late post-ICU attrition due to disease progression.
1-year mortality	50–70% (mean ≈ 62%)	Long-term survival is possible in one-third of ICU-treated oncology patients.
Return to oncologic therapy after ICU	28–45% of survivors	Indicates that one in three ICU survivors resumes active treatment, especially those with reversible complications.

#### 3.3.2. Mortality by Cancer Type (Table 8)

**Table 8:** Mortality by cancer type [18,22,23,25,54,64].

Cancer 1.	Median ICU Mortality	Median 1-Year Mortality	Notes
Hematologic malignancies	45–55%	60–65%	Highest early mortality; survival plateaus after day 7 if multiorgan failure avoided (de Vries 2019; Otten 2025).
Solid tumors	25–35%	50–55%	Better short-term outcomes; prognosis depends on metastatic status and performance score (Manz 2023; Praça 2024).
Mixed oncology cohorts	35–45%	55–60%	Intermediate outcomes; heterogeneity limits interpretation.
Postoperative or elective ICU	8–15%	25–30%	Clearly favorable subgroup.
Sepsis/infection-driven admissions	40–55%	60–65%	Similarly to non-cancer sepsis once adjusted for comorbidity.
COVID-19/viral pneumonia	60–80%	N/A	Mortality peaked 2020–2021; improved slightly post-vaccination (Bozdağ 2022; Šimkovič 2023).

#### 3.3.3. Impact of Organ Support (Table 9)

**Table 9:** Impact of organ support on mortality [2,23,24,25,35,36,42,44,45,68,70].

Organ	Associated Mortality/Prognostic Trend	Key References
Mechanical ventilation (IMV)	65–85% mortality; only 20–30% resume therapy	Otten 2025; Boldingh 2024; Saillard 2020
Renal replacement therapy (RRT)	Mortality > 80% if > 7 days; threshold for futility	de Vries 2019; Otten 2025
Vasopressors > 48 h	ICU mortality ≈ 70%	Nazer 2022; Fang 2017
Multiorgan failure (≥3 organs)	> 85% mortality	Storck 2025; Munshi 2021
Isolated single-organ dysfunction	15–30% mortality	de Vries 2019; Yang 2020
ECMO as bridge to therapy (e.g., CAR-T, targeted therapy)	Survival 50–70% in select cases	Luo 2025; Azoulay 2021

#### 3.3.4. Trends over Time (2015 → 2025)

ICU survival rates among oncologic patients have improved by approximately 8–10% over the past decade. The percentage of hematologic patients surviving to hospital discharge increased from about 40% before 2015 to over 55% after 2020, while the proportion of patients able to resume systemic cancer therapy nearly doubled—from roughly 20% to 40%.

These improvements mainly reflect earlier ICU admissions and quicker recognition of sepsis, the development of structured collaboration between oncology and critical care teams, and the increased availability of targeted and immune-based therapies. Together, these advances have reshaped the ICU’s role—from a setting mainly associated with end-of-life care to one that serves as an active therapeutic bridge supporting disease-modifying treatment and long-term survival.

#### 3.3.5. Quantitative Definition of “Benefit Likely” vs. “Futility Likely” (Table 10)

Quantitatively, about four in ten oncologic patients admitted to the ICU survive to discharge, and one in three resumes active cancer treatment. Mortality exponentially increases with the number of failing organs and disease refractoriness.

The survival inflection point occurs between two and three organ supports, confirming that time-limited ICU trials (5–7 days) can reliably distinguish between reversible and futile courses.

**Table 10:** Benefit vs. Futility.

Criterion	Typical Mortality Thresholds (Pooled Data)	Interpretation
Single-organ failure, controlled cancer	ICU mortality ≤ 30%	Benefit likely
Two-organ failure (IMV + vasopressors)	ICU mortality 50–60%	Context-dependent
≥ 3 organ supports or RRT > 7 days	ICU mortality ≥ 80%	Futility likely
Advanced/refractory malignancy + ECOG ≥ 3	1-year survival ≤ 15%	Futility likely
Postoperative/reversible toxicity (CAR-T, irAE)	ICU mortality ≤ 20%	Benefit likely

## 4. Discussion

This PRISMA Scoping Review confirms, with compelling clarity, that the prognosis susceptible to change through ICU admission is not dictated by the oncologic label per se, but by physiological reversibility and by the timing of the entire pathway: early triage, standardized diagnostic algorithms, proportional organ support, scheduled re-assessments, and early goal-concordant conversations. Decision-making should be anticipatory and oriented toward clinical windows of opportunity, not reactive or defensive [70].

### 4.1. Key Determinants of Benefit

In acute respiratory failure in the immunocompromised host, avoiding the “undetermined etiology” situation through a standardized admission work-up and multidisciplinary collaboration has been consistently associated with lower mortality and with a faster, safer return to antineoplastic therapy—a central message of contemporary syntheses [1]. Early ICU admission facilitates adequate oxygenation, minimally invasive diagnostic procedures, and timely calibration between escalation and de-escalation [70].

Accordingly, the decision to admit to ICU should be anchored in the probability of correcting a reversible physiology (refractory hypoxemia with a treatable cause, septic shock with feasible source control, potentially reversible toxicities of modern therapies) and in the clinical opportunity to preserve or resume oncologic intent. Assessment should be complemented by baseline performance status/frailty and by the number of failing organs at presentation [70].

Data synthesized by Azoulay and colleagues define the frame: respiratory events occur frequently in hematologic and solid tumor patients, and mortality increases when invasive mechanical ventilation is required, when the etiology remains unknown, when ICU admission is delayed, and when invasive fungal disease is present [1,70]. The recommended strategy is a “standardized admission diagnostic”: history and physical guided by the DIRECT mnemonic, blood cultures, sputum/induced sputum (including Pneumocystis), multiplex viral PCR, evaluation for cardiogenic pulmonary edema, and early high-resolution CT to raise pre-test probability and guide subsequent procedures [70].

In parallel, avoiding delayed intubation in patients with persistent respiratory drive (despite standard oxygen therapy/HFNC/NIV) remains a safety principle: the clinical response within the first 1–4 h should weigh decisively on whether to continue a non-invasive strategy versus intubate [70]. Joint assessment by pulmonology, infectious diseases, and hematology optimizes diagnostic yield and reduces exposure to therapeutic failure.

In brief, “the same rigor, the same speed” as in non-oncologic patients, but through the lens of the immunocompromised host: rapid estimation of risk for pathogens and non-infectious entities (e.g., checkpoint inhibitor pneumonitis, diffuse alveolar hemorrhage, drug-induced injury), early involvement of relevant specialties, bronchoscopy and BAL when the clinico-imaging signature calls for it (especially with suspected Pneumocystis), and preference for minimally invasive pathways with solid diagnostic productivity [70].

AGIHO/iCHOP 2019 is explicit: there is no evidence that sepsis/neutropenia in oncology should be resuscitated “differently” from the general population. The Sepsis-3/Surviving Sepsis imperatives remain intact: antipseudomonal broad-spectrum antimicrobials within the first hour, fluids, vasopressors as needed, blood cultures, lactate, and, above all, prompt source control [3]. Empirical antibiotics should be chosen according to local ecology (piperacillin/tazobactam or a carbapenem), with an aminoglycoside added in septic shock and de-escalation guided by cultures; early assessment of invasive fungal risk and initiation of antifungal therapy are indicated if instability persists [3].

The organizational component is equally decisive: early ICU admission in unclear situations, early warning scores on hemato-oncology wards, and daily ICU–oncology briefings for plan alignment and protocol execution—measures associated with reductions in mortality in consecutive cohorts [3]. Standardization of definitions and severity reporting (including ECOG/frailty) is essential for comparability and for designing prospective studies [3].

The literature aggregates four recurrent situations in which ICU benefit is probable: early admissions, single-organ failure, oncologic disease under control or on active treatment, treatable toxicities of modern therapies (e.g., checkpoint pneumonitis, cytokine-release syndromes), and the presence of standardized ICU protocols [70]. Conversely, refractory disease, severe functional decline (ECOG ≥ 3), and a cumulative need for organ support delineate zones with a low likelihood of patient-valued outcomes [70].

### 4.2. The Role of Time-Limited Trials (TLT)

Time-limited trials—based decisions—are instruments of proportionality: short windows (1–4 days) often suit solid tumors with an unfavorable prognosis, whereas in hemato-oncology or with less severe acute illness, longer windows (up to 8–12 days) may be justified before concluding that there is no benefit. [3] Explicit definitions of success/failure criteria and scheduled re-assessments (day 3–5) should be documented and communicated to the patient/family [4,5]. Palliative care integration as a transversal function—clarifying goals, setting expectations, and enabling compassionate de-escalation when futility signals converge—is now a standard recommendation in intensive care [5].

### 4.3. Lessons from the COVID-19 Era

The pandemic acted as a stress test for the ICU–oncology interface. Meta-analyses reported higher risks of mortality and ICU need in patients with cancer versus those without, yet no excess mortality in older adults ≥ 65 years—underscoring that triage must remain individualized, not “by diagnosis.” [9]. Organizational maturation (vaccination, dedicated flows, adaptive learning) progressively reduced risks, reinforcing the importance of synchronization and co-management [9].

### 4.4. Practical Implications for Triage and Co-Management Data

High-intensity resource use in the last month of life remains common and is associated with lower perceived quality of end-of-life care—further justification for early palliative integration and structured goals-of-care conversations [7]. Organizational and behavioral interventions (“nudges”) can increase timely conversations and better align intensity with patient preferences [5].

The expansion of targeted therapies and immunotherapy brings distinct toxicity profiles (pneumonitis, CRS, ICANS) that, if recognized early and treated in a standardized fashion, are frequently reversible [70]. In strictly selected cases and in experienced centers, advanced support (including ECMO) can serve as a bridge back to treatment, provided the oncologic trajectory is non-terminal [73].

After major oncologic surgery, “threshold” intensive monitoring (HDU/ICU) can prevent decompensation and readmission, particularly in high-risk patients (frailty, limited cardiopulmonary reserve). Population-based analyses in colorectal cancer suggest that appropriateness of level of care and transfer criteria influence hard outcomes (e.g., death/major complications), arguing for standardized pathways and audit [43].

### 4.5. Practical Synthesis

ARF algorithm in the immunocompromised host: DIRECT + minimal test panel at presentation; early CT; BAL/early pulmonology–infectious diseases input when indicated; avoid “undetermined etiology”; re-assess at 24–48 h; explicit criteria for early intubation when respiratory drive remains high under HFNC/NIV [70].

Sepsis/neutropenia: antimicrobials “within the first hour”, source control, hemodynamic management per standard; empirical antifungal therapy if instability persists; early ICU admission in unclear cases [3].

Time-limited trials: explicit success/failure criteria and horizon (1–4 vs. 8–12 days) according to tumor biology, injury severity, and the potential to resume therapy; ICU–oncology co-management; daily documentation and communication with patient/family [4,5].

Proportionality and EoL audit: intensity indicators in the last month of life and rates of goals-of-care conversations; integrate palliative care and, where appropriate, behavioral interventions (“nudges”) [5,7].

### 4.6. Limitations and Research Directions

The literature remains heterogeneous, and part of the evidence is observational, with selection risks (local ICU admission policies, variable thresholds for intubation and “do-not-intubate”). Pragmatic randomized studies are needed—on pathways (standardized diagnostic vs. usual care), on timing of intubation in hypoxemic ARF in the immunocompromised host, and on TLT models (windows, criteria, palliative integration) with patient-centered outcomes (function, return to oncologic therapy, quality of life) [70]. Future meta-analyses should disentangle disease effects from contextual effects (access, vaccination, system load) to support generalizable policies [9].

## 5. Conclusions

To sum up, this PRISMA Scoping Review shows that the value of ICU admission in oncology is not determined by the disease label, but by the reversibility of physiology and the timing of interventions: early triage, standardized diagnostics, proportional organ support, and scheduled re-evaluations. When acute respiratory failure in the immunocompromised host is rapidly elucidated and managed along clear pathways; when sepsis/neutropenia adheres—without derogation—to Sepsis-3/Surviving Sepsis principles; and when palliative care is integrated as a transversal function, the ICU becomes a genuine bridge back to oncologic intent. Clinical benefit concentrates in patients with preserved functional reserve and plausibly reversible injury, in contexts where time-limited trials set transparent criteria for continuation or de-escalation. The lesson of the pandemic confirms that triage must remain individualized, not “by diagnosis.” Stripped to essentials, the message is direct: for the right patient, at the right moment, with clear goals and disciplined implementation, ICU care can still change prognosis in oncology; conversely, in the absence of these conditions, intensity is unlikely to be proportional, and clinical honesty calls for realignment toward what truly matters to the patient.

## Supplementary Materials

The following supporting information can be downloaded at: https://www.mdpi.com/article/10.3390/cancers17223636/s1, Table S1: Overview of Studies Included in the Systematic Review.

## Abbreviations

The following abbreviations are used in this manuscript:

ACL F	Acute-on-chronic liver failure
AKI	Acute Kidney Injury
ALL	Acute Lymphoblastic Leukemia
AML	Acute Myeloid Leukemia
ANC	Absolute Neutrophil Count
APACHE II	Acute Physiology and Chronic Health Evaluation II
ARDS	Acute Respiratory Distress Syndrome
ARF	Acute Respiratory Failure
ART	Antiretroviral Therapy
aOR	Adjusted Odds Ratio
BCLC	Barcelona Clinic Liver Cancer classification
BFS	Bereaved Family Survey
BiTE	Bispecific T-cell Engager
CAR-T	Chimeric Antigen Receptor T-cell therapy
CCI	Charlson Comorbidity Index
CF S	Clinical Frailty Scale
CI	Confidence Interval
CLL	Chronic Lymphocytic Leukemia
COPD	Chronic Obstructive Pulmonary Disease
COVID-19	Coronavirus Disease 2019
CRC	Colorectal Cancer
CRP	C-reactive Protein
CRS	Cytokine Release Syndrome
CT	Chemotherapy
DLBCL	Diffuse Large B-cell Lymphoma
DNR	Do Not Resuscitate
ECOG	Eastern Cooperative Oncology Group
EFRAIM	European Federation of Critical Care Immunocompromised Patients
EOL	End of Life
FiO_2_	Fraction of Inspired Oxygen
G-CSF	Granulocyte Colony-Stimulating Factor
GI	Gastrointestinal
GU	Genitourinary
GCS	Glasgow Coma Scale
HCT	Hematopoietic Cell Transplant
HFNO	High-Flow Nasal Oxygen
HIV	Human Immunodeficiency Virus
HM	Hematologic Malignancy
HCC	Hepatocellular Carcinoma
HSCT	Hematopoietic Stem Cell Transplantation
HR	Hazard Ratio
ICANS	Immune Effector Cell-Associated Neurotoxicity Syndrome
ICI	Immune Checkpoint Inhibitor
ICU	Intensive Care Unit
IF I	Invasive Fungal Infection
IL-6	Interleukin-6
IMV	Invasive Mechanical Ventilation
IPC	Integrated Palliative Care
irAE	Immune-related Adverse Event
LOS	Length of Stay
MASCC	Multinational Association of Supportive Care in Cancer
MDS	Myelodysplastic Syndrome
MM	Multiple Myeloma
MOF	Multiple Organ Failure
MRD	Multidrug-Resistant
MV	Mechanical Ventilation
NHIRD	National Health Insurance Research Database (Taiwan)
NIPPV	Non-Invasive Positive Pressure Ventilation
NIV	Non-Invasive Ventilation
NSCLC	Non-Small-Cell Lung Cancer
OR	Odds Ratio
OS	Overall Survival
PCC	Population–Concept–Context
PEG	Percutaneous Endoscopic Gastrostomy
PS	Performance Status
PRISMA	Preferred Reporting Items for Systematic Reviews and Meta-Analyses
PRISMA-ScR	Preferred Reporting Items for Systematic Reviews and Meta-Analyses extension for Scoping Reviews
QoL	Quality of Life
qSOFA	Quick Sequential (Sepsis-related) Organ Failure Assessment
RCT	Randomized Controlled Trial
RRT	Renal Replacement Therapy
SAPS 3	Simplified Acute Physiology Score 3
ScR	Scoping Review
SCT	Stem Cell Transplantation
SIC	Serious Illness Conversation
SIRS	Systemic Inflammatory Response Syndrome
SOT	Solid Organ Transplant
SOFA	Sequential Organ Failure Assessment
SCLC	Small-Cell Lung Cancer
TLS	Tumor Lysis Syndrome
TPN	Total Parenteral Nutrition
VA	Veterans Affairs
WHO	World Health Organization
