# Supplementary material for: When and for Whom Does Intensive Care Unit Admission Change the Prognosis in Oncology?—A Scoping Review
Source: Cancers (Basel). 2025 Nov 12;17(22):3636. doi: 10.3390/cancers17223636 (PMC12650642; doi:10.3390/cancers17223636)
Supplement: Supplementary file 1 [file cancers-17-03636-s001.zip › cancers-3969627-supplementary.pdf]

TABLE S1: Overview of Studies Included in the Systematic Review

No.	Study ID (Author, Year)	Country, Study Design	Population (Cancer type, N, Age, Stage)	Reason for ICU Admission	Oncological Treatment Status	ICU Outcomes (ICU/Hospital Mortality)	Long-term Outcomes (Survival, QoL, Return to treatment)	Prognostic Factors Identified	Key Notes/Context
1	Aboueshia M, et al., 2021 [60]	USA (Tulane Univ. Hospitals, New Orleans, LA) Retrospective cohort (Feb–Apr 2020)	260 hospitalized COVID-19 patients; 57 with cancer (breast 21%, prostate 12%, lung 9%, AML 6%, others). Cancer pts older (mean 63.6 vs 58.7 yrs).	COVID-19 pneumonia /ARDS.	Active vs non-active cancer defined; mix of solid & hematologic malignancies. No details on therapies.	Overall hospital mortality 15.4% (40/260). No significant difference: cancer 12.3% vs non-cancer 16.3%. ICU admission: cancer 22.2% vs non-cancer 16.1% (NS). ICU mortality ~80% (vs ~19% on wards).	No structured long-term follow-up. LOS longer in cancer pts (12.8 vs 8.6 days). Active cancer subgroup had even longer LOS (24.9 vs 9.5 days, p<0.001).	Predictors of ICU admission: obesity (OR 4.5), smoking (OR 2.4), diabetes (OR 2.2), high qSOFA (OR 2.5). Predictors of mortality: obesity (OR 6.3), smoking (OR 6.1), CURB-65 (OR 2.8), qSOFA (OR 2.6). Cancer itself not associated with excess mortality.	Shows that comorbidities, not cancer diagnosis per se, drove COVID-19 ICU outcomes. Cancer patients did, however, stay longer in hospital and had higher re-intubation rates.
2	Alkan A, et al, 2024 [32]	Turkey (Hacettepe University, 2003–2020) Retrospective single-center cohort (17 years)	502 adult acute leukemia patients (AML 260, ALL 242). Mean age 48.3 years, 58% male. 30% underwent HSCT.	ICU admissions mainly due to IFI-related complications (sepsis, pulmonary failure, neutropenic infections). 38.6% of IFI patients required ICU.	Newly diagnosed, in remission, and relapsed AML/ALL included. Nearly all received chemotherapy; most received antifungal prophylaxis.	ICU follow-up increased risk of IFI (OR 2.5) and mortality (HR 2.49). Mechanical ventilation further increased mortality risk (HR 3.82). ICU patients had median survival 1 month vs 13 months without ICU admission.	Median survival with IFI: 5 months (range 1.9–8). Survival shorter in ICU patients (1 month) vs non-ICU (13 months). Only ~30% alive at study endpoint.	Risk factors: neutropenia (3.5-fold ↑ IFI), ICU admission, mechanical ventilation, mucormycosis infections had worst prognosis.	One of the largest single-center leukemia–IFI cohorts. Demonstrates ICU admission and MV as markers of very poor survival. Strong signal of futility in refractory IFI cases.

3	Azoulay E, et al, 2019 [1]	France (multicenter review) Narrative Review (state-of-the-art, Lancet Respir Med)	Immunocompromised adults: Hematologic malignancies (e.g., AML, ALL, lymphoma, MDS), solid tumors (lung cancer), solid organ transplant patients. No fixed N (review-based).	Acute respiratory failure (ARF), up to 50% of hematology patients and 15% of solid tumor patients.	Mixed: patients under active treatment, post-HSCT, and solid organ transplant recipients.	Mortality rates high: intubation/IMV and hospital mortality up to 40% when oxygen ≥6 L/min. Delayed ICU admission increases mortality.	Long-term outcomes not consistently reported; emphasis on acute survival.	Negative prognostic factors: invasive mechanical ventilation, multi-organ dysfunction, older age, frailty, poor performance status, delayed ICU admission, ARF due to fungal infection or unknown cause.	Landmark review article summarizing epidemiology, risk factors, diagnostic approach, and management of ARF in immunocompromised patients. Strong emphasis on early ICU admission and multidisciplinary approach.
4	Azoulay E, et al., 2021 (CARTTAS study, Nine-I network)[70]	Multinational (France, Spain, USA, UK, Russia, Canada, Germany, Austria; 21 ICUs) International multicenter observational cohort	241 patients admitted to ICU within 30 days of CAR T infusion (from 942 total treated). Adult patients with refractory hematologic malignancies (mainly B-cell lymphomas, ALL, MM). Median ICU admission 4.5 days post-infusion.	CAR T-cell toxicities: cytokine release syndrome (42%), CRS + ICANS (39%), ICANS alone (3%). Infections in 12%. Shock, ARDS, and neurotoxicity common.	All within 30 days of CAR T therapy for refractory disease.	90-day mortality 22.4%. Life-saving interventions within 24h in 31% (mainly vasopressors).	At 90 days, ~78% alive. Long-term survival linked to hematologic response but not systematically reported. ICU care allowed many to return to cancer remission pathways.	Independent predictors of 90-day death: frailty (HR 2.5), bacterial infection (HR 2.1), and need for life-saving therapy within 24h (HR 1.8). CRS/ICANS grade not independently predictive.	Demonstrates ICU is an essential bridge for CAR T patients, with majority surviving ICU and resuming remission. ICU admission is not futile — outcomes far better than for traditional sepsis/ARDS in hematology.

5	Belsky JA, et al., 2021 [61]	USA (systematic review of global data) Systematic review & meta-analysis (PRISMA-guided). Literature search until July 2020.	4,942 immunocompromised patients with COVID-19: 2,360 adult cancer (A-CA, mostly solid tumors), 653 hematologic malignancies, 623 pediatric cancer (P-CA), 30 HCT, and 1,018 SOT.	COVID-19 pneumonia /respiratory failure. ICU admission defined as ventilatory/ inotropic support.	Mixed: many on active treatment; delays in chemotherapy (56% A-CA, 28% P-CA) reported.	ICU/critical illness prevalence: 20.2% in adult CA, 32% in HCT, 43% in SOT. Mortality: A-CA 28.1%, P-CA 10.9%, SOT 23.2%. HCT mortality ~similar to general population.	Limited follow-up. Pediatric cancer patients had similar or better outcomes vs general population. No systematic QoL or return-to-treatment data.	Poorer outcomes in adult CA + SOT vs general population; HCT and pediatric cancer outcomes closer to general population. Higher comorbidities, CRP, IL-6, D-dimer linked to severity.	First large synthesis across CA, HCT, SOT. Shows heterogeneity: not all immunocompromised are equally high risk. ICU not always futile in pediatrics or HCT.
6	Boldingh JWHL et al., 2024 [24]	Netherlands (5 university hospitals; HEMA-ICU Study Group) Multicenter retrospective cohort with internal–external validation	1,097 hematologic malignancy patients (AML 35%, NHL 23%, myeloma 13%, others 28%); mean age 55 ± 15 years; 21% post-allo HSCT; 64% active disease.	Acute medical or surgical ICU admissions >24 h; sepsis 56%, respiratory failure 64%, renal failure 15%, shock/organ failure.	Mixed—new diagnosis, remission, relapsed, and post-transplant; all hospitalized adults.	ICU mortality ≈46%; hospital mortality 46%; 1-year mortality 62% (95% CI 59–65).	1-year survival 38%; no QoL or return-to-therapy data.	8 main predictors (from 13-variable model): mechanical ventilation, active disease, age, prior allo-HSCT, lowest platelet count, AKI, max heart rate, type of malignancy. C-statistic = 0.70 (95% CI 0.63–0.77), outperforming APACHE II (C = 0.61).	First validated long-term prognostic model for hematologic ICU patients. Predictive for 1-year outcome—useful for shared decision-making and time-limited ICU trials. Suggests critical benefit only for patients without mechanical ventilation, AKI, or refractory disease.

7	Bouteloup M, et al, 2017 [14]	France (GRRR-OH research group) Systematic review + meta-analysis (38 studies, 6,054 critically ill cancer patients, 2005–2015)	2,097 neutropenic patients; 3,957 non-neutropenic. Hematologic and solid tumors included.	ICU admission for mixed reasons: respiratory failure, sepsis, organ dysfunction. Neutropenia analyzed as a subgroup.	Mixed oncologic status: hematological malignancies, solid tumors, with/without active therapy.	Median mortality overall: 54%. Neutropenic patients: 60% mortality. Non-neutropenic: 49%. Pooled difference ~+10% (95% CI 6–14%).	Very limited long-term outcome reporting; only 1 study had 6-month follow-up. No consistent QoL or therapy resumption data.	Neutropenia linked to higher raw mortality, but after severity adjustment, not independently predictive. Survival improved over time in non-neutropenic patients, but not in neutropenic patients.	Main conclusion: neutropenia should not be used as a triage exclusion criterion. Despite worse crude outcomes, meaningful survival exists, especially if disease otherwise treatable.
8	Brown CE, et al., 2016 [71]	USA (15 hospitals, Seattle/Tacoma) Secondary analysis of multicenter trial (2002–2008)	829 ICU patients who died: 158 with metastatic cancer, 592 with COPD, 79 with ILD. Median age cancer 64, lung disease 72–73.	All patients died in ICU; reasons varied (respiratory failure, organ failure, sepsis). Study focused on palliative care elements.	Metastatic cancer group only (solid tumors, advanced stage). No active therapy details.	All died in ICU (100%). Compared elements of palliative care rather than survival.	Not applicable (all patients deceased). Focus on processes of care.	Cancer patients were more likely to: have DNR at death, have prognosis discussed, avoid CPR before death, have pain assessment. COPD/ILD patients had longer ICU stays and less documentation of palliative discussions.	Shows that cancer patients receive more structured palliative care in ICU than non-cancer chronic disease patients. Cancer diagnosis was historically associated with better prognostic discussions and end-of-life planning.

9	Carini L, et al., 2024 [67]	International (ICONIC network: Europe, South America, Asia, Africa) Prospective multicenter observational study (2018–2022)	1,400 patients with lung cancer (NSCLC 80%, SCLC 20%) admitted to 132 ICUs; median age 66; 63% male; 47% metastatic.	Sepsis (31%), ARDS/respiratory failure (28%), post-procedure or surgical (18%), cardiovascular failure (9%).	54% on active systemic therapy, 11% immunotherapy, 7% targeted therapy; 28% treatment-naïve.	ICU mortality 32%, hospital 42%, 6-month mortality 64%.	6-month survival 36%, return to oncologic therapy 48% of ICU survivors. Median post-ICU OS: 8.3 months (95% CI 6.1–10.5).	Independent mortality predictors: metastatic disease, performance status ≥2, invasive ventilation, AKI, vasopressors. Protective: curative intent therapy, immunotherapy.	Largest contemporary lung cancer ICU cohort. Demonstrates that nearly half of ICU survivors resume cancer therapy—definitively showing that ICU admission can change prognosis in selected lung cancer patients. Immunotherapy responders particularly benefited.
10	Chen W-C, et al, 2018 [31]	Taiwan Retrospective observational, single center (2005–2010)	58 lung cancer patients with ARF; mean age 76; 80% NSCLC, 20% SCLC; 72% stage IV.	Acute respiratory failure (hypoxemic 21%, hypercapnic 38%, mixed 41%).	62% progressive or newly diagnosed disease; most post-chemotherapy.	28-day mortality 39.7%; 90-day 63.8%; 1-year 86.2%. NIPPV failure common when used as first-line therapy.	1-year survival 13.8%; no QoL or functional data.	Independent predictors of 28-day mortality: (1) progressive/newly diagnosed lung cancer (OR 14.0), (2) NIPPV as first-line therapy (OR 35.4), (3) ≥2 organ failures (OR 18.1).	Outcomes poorest when NIPPV initiated as first-line therapy for cancer-related ARF. Post-extubation NIPPV associated with better survival. Early ICU admission before multi-organ failure crucial.

11	Chiang J-K, et al, 2019 [34]	Taiwan Nationwide population-based retrospective cohort (Taiwan NHIRD)	1,177 adult chronic dialysis patients who died between 2006–2011; 149 (12.7%) with cancer, 1,028 without; mean age 70 years. Common cancers: kidney/bladder (34%), liver (24%), colon (16%).	Not specified by cause; analysis of ICU admissions in last month of life.	Mixed; all at end-of-life (decedent cohort).	ICU admission: 51% overall (42% in dialysis + cancer vs 52% non-cancer, $p = 0.028$). CPR: 67% overall (47% vs 70%, $p < 0.001$). Hospital death: 65% overall (73% vs 64%, $p = 0.027$).	All-cause mortality by design; median survival from dialysis initiation ≈ 2.6 years. No QoL data.	Independent factors: Cancer associated with ↓CPR (OR 0.39), ↑>25 day hospital stay (OR 1.52), ↑≥2 hospitalizations (OR 2.26). ICU admission not significantly different after adjustment.	Cancer patients received more palliative care (11.4% vs 0.2%), fewer resuscitative interventions, similar costs, and longer hospitalizations — suggesting better recognition of prognosis and more appropriate EOL care. Highlights cultural and policy impacts on ICU use.
12	Civriz Bozdağ et al., 2022 [54]	Turkey (25 hematology centers) Multicenter retrospective registry (Mar–Nov 2020)	340 adults (and 5 pediatric) with hematological malignancies and SARS-CoV-2 infection. Median age 59 (7–93), M/F = 1.3. MM 25%, AML 20%, NHL 18%.	COVID-19-related acute respiratory failure, ARDS, or septic shock. 25% admitted to ICU.	28% active hematologic disease; 19% newly diagnosed; 21% had treatment modified due to pandemic. 14 auto-HSCT, 38 allo-HSCT.	ICU admission 25%; overall mortality 26.5%. Mortality by severity: mild 4.4%, moderate 12.4%, severe/critical 83%. Among allo-HSCT 23.7% died; auto-HSCT 21%.	No post-discharge data. Median PCR negativity 11 days.	Independent predictors of death (multivariate Cox): active hematologic disease (HR 2.4), neutropenia (HR 1.95), ICU admission (HR 3.05), intubation (HR 12.0), life expectancy <12 months (HR 2.1–2.5), and hydroxychloroquine use (HR 4.99 vs favipiravir).	Large national cohort — confirms COVID-19 lethality in hematologic malignancy ICU patients. Demonstrates disease activity, neutropenia, and ICU admission as key mortality drivers. HCQ exposure associated with higher death risk. Favipiravir appeared safer.

13	Dale CD, et al, 2016 [43]	Scotland, UK (West of Scotland, population-based registry) Multicenter retrospective observational (linked cancer registry + ICU database, 2005–2011)	You could cite it in your Discussion under "novel oncology-derived prognostic indices may also stratify ICU outcomes," but it wouldn't fit in your main Results dataset (since your inclusion criteria required cancer patients).	Postoperative complications requiring ICU organ support: sepsis, anastomotic leak, cardiorespiratory failure. 9% required ICU organ support.	Both elective (73%) and emergency (27%) CRC surgeries included. Patients had operable disease; metastatic patients less likely to receive ICU support.	Hospital mortality: overall 6%. In patients requiring ICU organ support: 28% vs 3% without organ support. ICU organ support group accounted for 48% of all in-hospital deaths.	6-month mortality: 35% in those requiring organ support vs 8% without. Survival significantly worse if organ support required.	Risk ↑: age ≥65 (OR 1.37), male sex (OR 1.46), emergency surgery (OR 3.45), congestive cardiac failure (OR 2.07), type II diabetes (OR 1.49), social deprivation. Risk ↓: metastatic disease (OR 0.61) and higher socioeconomic status.	~1 in 10 CRC surgical patients need ICU organ support. ICU organ support patients had nearly 10-fold higher hospital mortality. Notably, metastatic patients were less likely to receive ICU support, likely due to selective admission policies (futility considerations).
14	de Freitas ICL, et al, 2020 [16]	Brazil (4 ICUs: São Paulo, Barretos, Porto Alegre, São Luís) Multicenter retrospective cohort (2009–2017)	226 patients with esophageal cancer, median age ~62, 77% male. Histology: squamous cell carcinoma > adenocarcinoma. 55% had metastatic disease.	Sepsis/septic shock (39%), acute respiratory failure (29%), other acute complications.	Advanced disease predominant; over half metastatic. Mix of emergency medical and urgent surgical admissions. Elective surgery excluded.	In-hospital mortality 58% (131/226). ICU mortality ~similar (not separated). Mortality in mechanically ventilated: 76%.	No structured long-term survival reported. Median LOS: survivors 13 days hospital vs non-survivors 3 days. No return-to-treatment data.	Independent mortality predictors: mechanical ventilation (OR 6.2) and metastatic disease (OR 7.1). High SAPS 3, high SOFA also associated.	Shows ICU mortality in unplanned esophageal cancer admissions is very high. Especially futile with mechanical ventilation and stage IV disease. Supports selective ICU admission based on disease stage and acute condition.

15	de Vries VA, et al., 2019 (HEMA-ICU Study Group) [25]	Netherlands (5 university hospitals) Retrospective multicenter cohort (2002–2015)	1,097 patients with hematologic malignancies: AML 35%, NHL 30%, MM 13%, ALL 7%, HL 4%, others. Mean age 55, 63% active disease.	Sepsis (50%), disease-related (18%), hemorrhage (7%), neurologic (7%), pulmonary embolus, perforation, treatment toxicity.	~21% had allogeneic HSCT; 7% autologous. Majority with active/relapsed disease.	28-day survival 56%, 3-month 48%, 1-year 38%. Mortality strongly linked to number/type of organ failures: 1 organ ~38% survival, 2 organs 27%, 3 organs 22%, 4 organs 8%.	WHO performance status at 3 months available in 493 survivors: 39% had good PS (0–1). Function not correlated with number of failing organs.	Respiratory failure (need for MV) strongest negative predictor. Active disease, low platelets, hepatic/renal failure, and high APACHE II also worsened prognosis. Some subtypes (ALL, HL, MM) had better 1-year survival than AML.	Key conclusion: Even with multiple organ failure, meaningful survival (and good function) is possible. MOF should not be an exclusion criterion for ICU admission in hematologic malignancy patients.
16	dos Reis AM, et al., 2017 [46]	Brazil (Porto Alegre, University Hospital) Prospective cross-sectional study	130 ICU patients: 65 critically ill with solid tumors (GI 54%, lung 14%, GU 14%, breast/cervical 5%, thyroid 2%), 65 non-cancer controls. Mean age 62, 57% male. 30% post-op oncologic patients.	Mixed: sepsis (28%), postoperative complications, organ failure.	Solid tumors only; hematologic malignancies excluded. Mix of recent surgery, active cancer, or progressive disease.	ICU mortality overall 44%. In cancer group, 28% (18/65) died vs 57% in controls. Median ICU stay to death ~11 days.	3-month follow-up showed sustained mortality differences, but no long-term functional or therapy resumption outcomes reported.	Independent mortality predictors (multivariate): high serum magnesium (OR 3.97), presence of solid tumor (OR 2.68), sepsis (OR 3.2). Absence of sepsis protective (OR 0.31). Age, comorbidities, BMI not significant.	Early biochemical markers (esp. electrolytes) can help identify oncology ICU patients at higher risk of death. Suggests lab-based triage may complement clinical severity scores.
17	Ediboğlu Ö, et al., 2018 [47]	Turkey (Izmir + Istanbul, pulmonary specialty ICUs) Retrospective cohort (2008–2015)	583 adult cancer patients: 472 lung cancer (81%), 111 extrapulmonary malignancies (19%). Median age 64, 82% male.	Respiratory failure: mostly direct tumor invasion (67%), infection (16%), others (17%). 78% required invasive mechanical ventilation.	Advanced and metastatic disease predominated. Both lung and extrapulmonary cancers included; many late-stage.	ICU mortality 53% (311/583). Hospital mortality not separately reported.	1-year mortality 80%. Median survival after ICU admission 12 days. Very few long-term survivors.	Independent risk factors: female sex (OR 2.07), need for intubation/IMV (OR 2.84), higher APACHE II score (OR 1.10 per point). Age/comorbidities not significant.	Demonstrates very high short- and long-term mortality in advanced-stage cancer ICU patients with respiratory failure. IMV is especially associated with futility.

18	El-Hibri F, et al, 2024 [55]	UK (Academic ICU, National Centre for HIV Malignancy, 2007–2020) Retrospective observational cohort	106 lymphoma patients: 63 HIV-associated, 43 non-HIV lymphoma. Mean age 46 (HIV-lymphoma) vs 66 (lymphoma alone). Predominantly advanced stage (92% stage IVB in HIV group).	Common ICU admission triggers: cardiovascular, respiratory, hematologic/immune complications. HIV-lymphoma more often admitted for hematologic/immune issues.	All treated with standard intensive chemotherapy regimens; ART in HIV patients. Mix of Burkitt, DLBCL, Hodgkin.	ICU survival: 71% HIV-lymphoma vs 72% lymphoma-alone. Hospital survival 44% HIV-lymphoma vs 58% lymphoma-alone. No statistically significant differences.	5-year survival ~30% in both groups; no significant difference between HIV+ and HIV-lymphoma patients.	Predictors of worse ICU outcomes: emergency admission, higher APACHE II, higher lactate, number of level 3 support days. For long-term survival: mechanical ventilation and higher APACHE II predicted worse 5-year outcomes.	HIV status does not affect ICU or long-term outcomes in lymphoma. Severity of illness and organ support are the major determinants. Contradicts old assumptions that HIV-related lymphoma ICU patients fare worse.
19	Elkrief A, Desilets A, Papneja N, et al., 2020 [57]	Canada (6 academic cancer centers, Quebec + BC) Multicenter observational cohort (Mar–Jun 2020)	252 cancer patients with COVID-19; 108 (42.9%) hospital-acquired. Median age ~72, mix of solid (lung, breast, GI, GU) and hematologic cancers.	COVID-19 pneumonia/respiratory failure. ICU admission rates not detailed separately, but nosocomial patients had higher severity.	44% on active treatment; included chemotherapy, targeted agents, immunotherapy, radiotherapy.	Mortality significantly higher in hospital-acquired cases (49.4%) vs community-acquired (22.9%). Nosocomial COVID independently predicted worse outcomes (HR ~2).	No long-term follow-up beyond acute episode.	Independent predictors of severe outcome: hospital-acquired infection, older age, hematologic malignancy, ECOG ≥2.	One of the first studies to highlight nosocomial COVID-19 in oncology patients. Shows that ICU-level severity and mortality were markedly higher in this group.

20	Epstein AS, et al., 2020 [51]	USA (Memorial Sloan Kettering Cancer Center, New York) Retrospective cohort (single center, 2014 admissions)		Sepsis (39%), acute respiratory failure (29%), other progressive complications (32%). Excluded: post-op complications, GI bleeding, chemo hypersensitivity.	All patients had progressive disease beyond first-line therapy, refractory to systemic treatment.	ICU mortality: 36% (10/28). Hospital mortality: 47% (13/28). Only 54% (15/28) discharged alive.	Median survival after discharge: 68 days (2.2 months). At 3 months, 40% alive; at 6 months, only 13% alive. 6 patients received further chemotherapy — none showed clinical benefit. No effective benefit from liver-directed therapies either.	Worse outcomes linked with: vasopressor use (80% in non-survivors vs 32% in survivors), intubation on admission, admission from medical floor. Overall, refractory disease status itself predicted futility.	Clear evidence that ICU admission in progressive metastatic GI cancer refractory to treatment does not alter prognosis or enable meaningful return to oncological therapy. Strong argument for shifting to palliative/supportive care.
21	Ersek M, et al., 2017 [38]	United States (VA Health System, 128 centers) Retrospective observational cohort using linked VA + Medicare + Bereaved Family Survey data	847 veterans with stage IV NSCLC who died in VA inpatient facilities (2010–2012). Mean age ~70; 34% < 65 years; 98% male.	"Aggressive care" = chemo or mechanical ventilation, ≥ 2 acute hospitalizations, or ICU admission within last 30 days of life.	31% received chemotherapy in last month of life; 25% ICU; 67% ≥ 2 hospitalizations.	N/A – all patients deceased; 72% had ≥1 aggressive episode in final 30 days.	Bereaved Family Survey (BFS): overall "excellent care" rating lower with any aggressive care (55.7 vs 67.6%; p = .002). Respectful Care & Communication factor lower (13.0 vs 13.8/15; p < .001). Hospice/palliative unit deaths mitigated this negative association.	↓ Family-rated quality associated with chemo (adj OR 0.52), ≥ 2 hospitalizations, or ICU admission. Hospice care buffered negative effects. Age not associated with aggressiveness.	Aggressive end-of-life care common (72%) despite limited benefit. Family-perceived quality poorer when care was intensive, but mitigated in palliative/hospice units. Supports early integration of palliative care and avoidance of futile ICU admissions at end-of-life.

22	Fang WF, et al., 2017 [44]	Taiwan (Kaohsiung Chang Gung Memorial Hospital, 3 ICUs) Combined retrospective + prospective observational (2013–2016)	532 sepsis patients; 95 with active cancer (18%). Active cancer: lung (20%), head/neck (18%), hematologic (14%). Median age 62 vs 67 in non-cancer.	Severe sepsis/septic shock (per Sepsis-3). >90% required mechanical ventilation.	78% with inoperable/recurrent cancer; 22% post-surgical without recurrence ("inactive cancer").	ICU mortality: higher in active cancer vs no cancer. 14-day 38.9% vs 18.5%; 28-day 50.5% vs 25.2%; 90-day 66.3% vs 36.6% (all p<0.001).	No structured long-term QoL or therapy return, but clear 90-day survival disadvantage in active cancer.	Higher baseline IL-10 levels in active cancer → predicted 14/28/90-day mortality. Higher trend of G-CSF also linked to worse survival. Shock status modified impact (in septic shock, mortality similar with or without cancer).	First study to link immune biomarkers (IL-10, G-CSF) with excess mortality in cancer sepsis ICU patients. Shows cancer-specific immune dysfunction worsens outcomes beyond standard severity scores.
23	Fletcher SA,et al., 2016 [72]	USA Retrospective population-based (SEER–Medicare, 2006–2011)	6,955 patients ≥65 y with myelodysplastic syndromes (MDS); 58% male, 84% White. 59% chemo-naïve before last 30 days of life.	ICU admission in last 30 days of life considered indicator of intensive EOL care.	41% received chemotherapy at some point; 23% were transfusion-dependent in last month of life.	28% ICU admission in last 30 days; 7% chemo within 14 days of death; 49% hospice enrollment. No direct ICU mortality reported (claims data only).	No QoL or survival follow-up (all decedents). Median hospice duration > 3 days for 78% of enrollees.	Higher odds of ICU admission: transfusion-dependence (OR 1.80), non-White race (OR 1.19), higher comorbidity. Lower hospice use: transfusion-dependence (OR 0.69), non-White race (OR 0.77), male sex (OR 0.78).	Demonstrates variable but potentially suboptimal EOL care in MDS. ICU admission may not always represent "futile" care due to reversible marrow failure and sepsis. Argues that current hospice models (which exclude transfusions) may limit appropriate palliative options for MDS.

24	Fortuny M, et al., 2025 [52]	20 centers worldwide (Europe, Latin America, Asia) Multicenter retrospective cohort (2012–2024)	1065 advanced HCC patients treated with ICI; 47 (4.4%) admitted to ICU. Median age 67, 77% male, 94% cirrhotic, 78% BCLC-C, 60% first-line ICI.	Immune-related adverse events (irAEs, 47%), variceal bleeding (30%), other cirrhosis-related complications.	All patients on ICI (atezolizumab-bevacizumab 68%, nivolumab 13%, durvalumab/tremelimumab 11%, others). ~60% first-line systemic therapy.	ICU mortality 25.5% (12/47). Two-thirds alive 28 days after ICU discharge. 3-month survival 83%, 6-month survival 69%.	Nearly half of survivors (48%) resumed immunotherapy or started new HCC therapy after ICU. Median time to restart ~17 days post-discharge.	Higher SOFA score predicted ICU, 28-day, and 6-month mortality (AUC 0.84). ACLF associated with worse outcomes (57% vs 24% mortality). irAE type influenced short-term mortality (neurological/cardiac worse).	First international HCC–ICI ICU study. Shows ICU care can meaningfully extend survival and allow continuation of therapy in selected advanced HCC patients — contrary to historical ICU exclusion.
25	Giannakoulis VG, et al, 2020 [9]	Greece (multicontinental dataset: Asia, Europe, USA) Systematic review & meta-analysis (PROSPERO CRD42020181531)	32 observational studies; 46,499 COVID-19 patients, including 1,776 with cancer. Median ages 42–74.	COVID-19 pneumonia /severe disease requiring ICU or invasive ventilation.	Mixed solid and hematologic cancers; treatment details not consistently reported.	All-cause mortality higher in cancer vs non-cancer (RR 1.66, 95% CI 1.33–2.07, *p*<0.0001). ICU admission more likely in cancer (RR 1.56, 95% CI 1.31–1.87, *p*<0.0001).	In subgroup ≥65 yrs (8 studies, n=5,438), mortality comparable (RR 1.06, 95% CI 0.79–1.41). No long-term outcomes reported.	Cancer independently increased risk of ICU admission and death, but effect attenuated in elderly.	Among the first meta-analyses quantifying risk: concluded that cancer confers ~60% higher risk of ICU admission and mortality from COVID-19, except in very old patients where baseline risk dominates. Highlights uncertainty about futility of ICU in this group and heterogeneity by age and cancer type.

26	Guarneri V, et al., 2021 [26]	Italy (Veneto Oncology Network, 26 centers) Regional registry, observational study	170 cancer patients with PCR-confirmed SARS-CoV-2. Median age 70 (25–92). Most frequent cancers: breast (24%), GI (23%), GU (13%), lung (11%), hematologic (8%). 59% stage IV. 79% ≥1 comorbidity.	COVID-19 pneumonia and complications. 78% symptomatic, 6% ICU admission, 17% non-invasive ventilation or intubation.	52% on active anticancer treatment at infection: chemo (27%), targeted (14%), endocrine (11%), immunotherapy (5%). Treatments were interrupted in 68% of cases.	Overall mortality 33% (57/170). 17% directly from COVID-19. ICU admission carried very high odds of death (OR ~9).	No structured long-term follow-up, but ~40% of those who stopped anticancer therapy resumed it. Median viral clearance time 23 days.	Predictors of death: ECOG ≥2 (OR 4.3), age (per year OR 1.03), ≥2 comorbidities (OR 2.0), dyspnea (OR 6.0), severe COVID phenotype (OR 6.0), ICU admission (OR 9.1), neutrophil/lymphocyte ratio ≥7 (OR 7.7), thrombocytopenia (OR 2.8). Active cancer therapy was not associated with worse outcome.	Shows cancer patients had equal ICU access as non-cancer peers in Veneto, unlike other regions. Highlights need for nuanced triage: performance status and acute severity dominate prognosis, not tumor stage or treatment.
27	Gupta A, et al., 2022 [62]	India Single-centre prospective observational study (Sanjay Gandhi PGIMS, Lucknow; Mar 2020–May 2021)	242 patients with hematological malignancies and confirmed COVID-19; median age 41 yrs (3–83); 48% acute leukemia, 19% NHL, 15% myeloma; 31% pediatric.	Moderate–severe COVID-19 (n=89, 36.8%) with hypoxia/ARDS; mild infection (63.2%) often managed at home.	80% on active therapy (51% chemo, 16% chemo-immuno, 10% oral targeted, 2.5% post-SCT immunosuppression); 61% active disease, 39% in remission.	ICU mortality 53.9% among severe cases; overall 28-day mortality 13.3%, 56-day 19.8%. Invasive ventilation required in 42.7%.	56-day follow-up; no QoL data; survivors with mild/moderate disease resumed treatment after recovery.	On multivariate analysis, poor outcomes were linked to: severe COVID-19 (HR 1.8), secondary bacterial/fungal infection (HR 2.1), and invasive mechanical ventilation (HR 2.3). ALC ≥ 1500/µL was protective.	Among 8 post-transplant patients, 75% had severe disease; secondary sepsis carried 84% mortality. Authors emphasize early detection and ICU admission rather than delay until multi-organ failure.

28	Haider SF, et al, 2023 [12]	UK (multidisciplinary team, narrative review) Narrative review of literature + guidelines	Hematological malignancies broadly (AML, ALL, lymphoma, MM, CLL, etc.). No fixed N (review-based).	Sepsis, acute respiratory failure, tumor lysis syndrome, bleeding, thromboembolism, CAR-T toxicities.	Patients at all stages: newly diagnosed, undergoing chemotherapy, HSCT, novel therapies.	Summarizes literature: ICU admission common (13.9% within 1 year of diagnosis). In-hospital mortality ~31% for HM ICU patients; acute respiratory failure mortality ~44%; MV >50%.	1-year mortality ~62% (from Dutch cohort cited). Functional outcomes poor in ~60% (ECOG 2–4). No direct follow-up data (review-based).	Key adverse prognostic factors: multiple organ failure, respiratory failure, late initiation of organ support (>72h after admission), NIV failure. Better prognosis in CAR-T toxicities with timely therapy.	Practical synthesis for ICU teams. Strongly endorses ICU trials/time-limited ICU support. Highlights role of novel therapies (CAR-T, BiTEs, ICIs), sepsis management, TLS prevention, and need for early palliative care triggers.

29	Hajjar et al., 2019 (VANCS II Trial)[10]	Brazil (Instituto do Câncer do Estado de São Paulo, University of São Paulo) Single-center, double-blind randomized clinical trial + meta-analysis	250 adults with *solid or hematologic malignancy* and septic shock. Median SOFA 7; ~25% had chemotherapy ≤4 weeks before ICU admission; GI tract most common primary site.	Septic shock (infection + vasopressor requirement).	25% had recent chemotherapy (<4 weeks); remainder mixed remission or palliative status.	28-day mortality: Vasopressin 56.8% vs Norepinephrine 52.8% (p=0.52). 90-day mortality: 72% vs 75% (p=0.56). No difference in SOFA score, ventilator-free days, RRT use, or adverse events.	No data on post-ICU outcomes.	None statistically significant for vasopressor type. High baseline SOFA and MDR infection associated with mortality, but not intervention-dependent.	Vasopressin not superior to norepinephrine as first-line vasopressor in oncologic septic shock. Mortality ~55%, markedly higher than non-cancer septic shock cohorts. Suggested SOFA may underestimate mortality risk in malignancy-related sepsis. Meta-analysis showed vasopressin may reduce RRT requirement (RR 0.46) but not survival.

30	Kochanek M, et al., 2018 [3]	Germany (DGHO/iCHOP guideline group) Evidence-based guideline (systematic review + expert consensus)	Neutropenic cancer patients (hematologic & solid tumors) with sepsis/septic shock during chemotherapy. No fixed N; guideline covers adult population.	Sepsis and septic shock in the setting of chemotherapy-induced neutropenia.	Patients on intensive chemotherapy, HSCT, immunotherapy with neutropenia.	Not applicable — guideline document, but notes mortality in neutropenic sepsis often >50% in older cohorts, now improving with structured ICU management.	Not applicable.	Risk factors for sepsis/mortality: ANC <500/μL >7 days, pneumonia, hypophosphatemia, lactate >3 mmol/L, high procalcitonin, MASCC risk index <21, fungal infection, vasopressor need, organ failure. NIV failure strongly predictive of poor outcome.	Key recommendations: (i) early ICU admission (no delay if sepsis suspected), (ii) full-code ICU support if long-term survival possible, (iii) early broad-spectrum antibiotics (pip-tazo, meropenem, imipenem), (iv) antifungal therapy if high risk, (v) cautious transfusion (Hb <7 g/dL, platelets ≤10×10^9/L), (vi) avoid routine G-CSF/GM-CSF, (vii) NIV not recommended if PaO_2/FiO_2 <150, HFNO can be tried, early intubation if NIV fails.

31	Kruser M, et al, 2017 [39]	USA (Northwestern University, Chicago) Retrospective cohort (single tertiary center, 2010–2015)	1,424 patients with metastatic cancer admitted to ICU; 161 (11.3%) had received palliative RT within 12 months.	Common ICU admissions: sepsis, respiratory failure, hemodynamic collapse. Specific breakdown not provided.	All metastatic, palliative-intent disease; 11% with recent palliative RT.	In-hospital mortality: 36.7% in recent palliative RT group vs 16.6% in other metastatic cancer patients (p<0.001). Adjusted OR 2.08 (95% CI 1.34–3.21).	Very poor trajectory: only 21% of palliative RT patients received additional cancer-directed therapy after ICU admission. Most did not return to oncological treatment.	Recent palliative RT predicted worse ICU survival, independent of acute illness severity.	Strong signal of futility: metastatic patients with recent palliative RT rarely survive ICU or return to cancer care. Supports role of early palliative goals-of-care discussions.
32	Lara OD, et al., 2020 [58]	USA (NYC, 6 hospital systems) Retrospective, multicenter cohort	121 gynecologic cancer patients with COVID-19 (median age 64, IQR 51–73). Ovarian (27%), endometrial (39%), cervical (11%), others. 63% advanced stage.	COVID-19 infection, pneumonia, and respiratory failure.	69% with active disease; 57% undergoing treatment at diagnosis (chemo, targeted therapy, immunotherapy, radiation, surgery).	Hospitalization: 66/121 (55%). ICU admission: 20/66 (30%). Invasive mechanical ventilation: 9/20 (45%). ICU mortality: 100% in ventilated patients. Overall COVID-19 case fatality: 14%. Hospitalized fatality: 25.7%.	No long-term outcomes beyond acute hospitalization.	Hospitalization associated with age ≥64 (RR 2.22), Black/African American race (RR 2.22), ≥3 comorbidities (RR 2.01). Death associated only with recent immunotherapy use (RR 2.93).	First large gynecologic cancer–specific COVID study. Major finding: no gynecologic cancer patient intubated for COVID-19 survived, highlighting futility of IMV in this setting. Cytotoxic chemotherapy and recent surgery did not increase risk of death.

33	Lara OD, et al., 2022 [27]	USA (8 hospital systems, New York City) Multicenter retrospective cohort	193 patients with gynecologic cancer and COVID-19 (ovarian 41%, endometrial 30%, cervical 17%); median age 65 (IQR 53–73).	COVID-19 pneumonia; 39 (36.8%) required ICU, 13 (12.3%) mechanical ventilation.	49.2% not on active treatment at diagnosis; 27% had chemotherapy within 90 days; 10% hormonal therapy; 5% immunotherapy.	Overall mortality 17.6%. No patient requiring mechanical ventilation survived. ICU mortality ≈33%.	Not reported (pandemic-era short follow-up).	Hospitalization: ≥65 yrs (OR 2.12), Black race (OR 2.53), PS ≥2 (OR 3.67), ≥3 comorbidities (OR 2.00). Mortality: only smoking (OR 2.75) significant. Chemotherapy/immunotherapy not associated with worse outcomes.	Demonstrates that demographics, comorbidities, and performance status—not oncologic treatment—determine outcomes. Suggests continuation of cancer care feasible with precautions. ICU mortality extremely high when invasive ventilation required.
34	Loh KP, et al., 2018 [19]	USA (California, statewide database) Retrospective observational cohort (2010 California SID)	5,862 admissions with metastatic cancer receiving ≥1 critical care therapy (CCT). Common cancers: lung (14.1%), colorectal (13.9%), genitourinary (7.6%), breast (4.6%). Mean age 66, 50% male.	Invasive mechanical ventilation (51.8%), TPN (37.5%), PEG (14.1%), tracheostomy (8.4%), dialysis for AKI (8.1%). Principal diagnoses: cancer-related (48%), infections (19%), pulmonary	All metastatic disease; incurable stage, mix of lung, breast, colorectal, GU.	Overall in-hospital mortality: 36.5%. Mortality higher with IPC use (63.9% vs 29.8%, reflecting selection of sicker patients). Among IMV subgroup, mortality 57% overall, rising to 82% with IPC involvement.	No long-term follow-up beyond discharge. Discharge destination: ~66% of survivors to home, 26% to facility. IPC associated with lower cost and shorter LOS among survivors.	Predictors of IPC use: lung cancer, higher comorbidity, documented DNR, infections, large hospitals. Mortality predictors: IMV, multiple CCTs, higher severity.	Demonstrates extremely poor outcomes in metastatic cancer patients requiring invasive CCTs. IPC use low but associated with resource optimization. Highlights futility of IMV in this population.

				disorders (9%).					
35	Luo X, et al., 2025 [68]	China (Guangzhou, multicenter contributors) Case series (3 patients) + comprehensive literature review	3 patients with newly suspected or confirmed lung adenocarcinoma. Ages 40, 64, 67. All presented with severe ARF refractory to ventilation.	Acute respiratory failure due to diffuse lung lesions, often undiagnosed at ICU entry.	No prior confirmed diagnosis at admission; ECMO enabled biopsy & genotyping (ROS1+, EGFR exon19+, KRAS G12V).	ICU survival: 2/3 survived, successfully weaned from ECMO (14–21 days). 1/3 died due to uncontrolled infection + tumor progression.	Survivors resumed targeted therapy (crizotinib, furmonertinib) and had marked recovery; one patient remained self-sufficient 1 year later.	Predictors of benefit: actionable mutations (ROS1, EGFR), feasibility of biopsy under ECMO, preserved organ function. Predictors of futility: absence of targetable mutations, uncontrolled infection, PS 4.	First series showing ECMO can be justified in advanced lung cancer if it bridges to targeted therapy. Demonstrates safety of biopsy under ECMO. Calls for individualized decisions instead of blanket futility.
36	Manz CR, et al., 2023 [22]	USA (University of Pennsylvania Health System, 9 oncology clinics) Stepped-wedge randomized clinical trial (machine learning + behavioral nudges)	20,506 cancer patients (41,021 encounters). Mean age 60. Mix of solid and hematologic malignancies; 13.5% high-risk (≥10% predicted 6-month mortality).	Not ICU-based; focused on outpatient end-of-life and prognostic discussions, indirectly affecting ICU use.	Mixed—outpatients with active or advanced disease under ongoing treatment.	End-of-life ICU use unchanged (15–17%). Systemic therapy within 14 days of death decreased from 10.4% to 7.5%. Inpatient deaths unchanged (~12%).	No QoL or survival data collected, but likely improved goal-concordant care and reduced overtreatment.	Intervention increased SICs (3.4% → 13.5%; aOR 2.62). Reduced end-of-life chemo (aOR 0.25). No change in ICU admissions or inpatient deaths.	Machine learning–triggered clinician nudges effectively reduced aggressive end-of-life care without limiting ICU access, promoting earlier goals-of-care planning.

37	Margolis B, et al., 2017 [8]	USA Retrospective population-based cohort (SEER–Medicare, 2000–2011)	5,873 women ≥65 years who died from uterine cancer. 47.6% endometrioid, 30.2% stage IV, 79.9% White.	ICU admission or ≥2 hospitalizations in last 30 days of life (proxy for high-intensity care).	Majority had advanced or metastatic disease; ~6.6% received chemo in final 14 days.	18.3% ICU admission, 15% ≥2 hospitalizations, 9% >14-day hospitalization, 42.5% high-intensity care overall.	Not applicable (all decedents).	Predictors of high-intensity care: younger age, Black race, ≥2 comorbidities, stage IV disease, Eastern US residence, recent diagnosis.	Median last-month Medicare cost: $7,645; 4× higher for high-intensity care ($16,173 vs $4,099). Rates of aggressive care stable over decade.
38	Marzorati C,et al., 2017 [15]	France & Belgium (17 ICUs, GRRR-OH network) Prospective multicenter cohort (post hoc analysis of 1011 hematologic ICU patients, 2010–2012)	226 adults (median age 63, 58% male) with hematologic malignancy and neurological failure. Most common cancers: NHL (37%), AML (21%), myeloma (10%), ALL/CLL (15%).	Neurological failure — drowsiness (65%), coma (33%), weakness (26%), seizures (19%). Causes: metabolic (32%), CNS infection (5%), vascular (6%), shock-related (23%).	42% had recent chemotherapy (<30 days); 9% autologous SCT; 8% allogeneic SCT; 22% in remission.	Hospital mortality 49.6%; 30-day 53.6%; 1-year 58%.	1-year mortality similar to patients without neuro failure (aHR 1.12, NS).	↑ Mortality: poor performance (OR 3.99), NHL (OR 2.60), shock (OR 1.95), respiratory failure (OR 2.18), low GCS (per point OR 0.88). ↓ Mortality: autologous SCT (OR 0.25).	Neurological failure was common (22%) and often multifactorial. Mortality high but not absolute—autologous SCT patients had survival benefit, suggesting selective reversibility. Study supports multidisciplinary triage and early, full-code ICU admission for potentially reversible neuro causes.

39	Michels G, et al, 2023 [5]	Germany (10 medical societies consensus) Consensus guideline / perspective (2018–2023)	Not original patient cohort. Applies to critically ill patients (oncologic and non-oncologic) requiring ICU, with emphasis on life-limiting disease and symptom burden.	Broad ICU scenarios: respiratory failure, cardiac disease, terminal renal disease, delirium, hematologic/oncologic disease, palliative emergencies.	Not applicable (guideline recommendations, not specific treatment cohorts).	Mortality not directly measured; consensus states ICU mortality remains unchanged with palliative integration, but QoL and satisfaction improved.	Long-term outcomes not numerically reported; recommendations focus on QoL, symptom control, patient/family communication, and decision-making.	Prognostic considerations: frailty (use of Clinical Frailty Scale), disease stage, therapy goals, patient's will, lack of benefit from invasive support (ventilation, dialysis, etc.).	Provides structured recommendations for timely palliative care integration in ICU. For oncology: stresses advance care planning, time-limited ICU trials, early multidisciplinary discussions, discontinuation of futile ICU care when prognosis is poor.
40	Munshi L, et al., 2021 [2]	Multinational (62 ICUs, 16 countries, EFRAIM dataset) Secondary analysis of prospective multinational cohort	801 patients with HM and ARF: 570 no-HCT, 86 autologous HCT, 145 allogeneic HCT. Median age 57–65. Common HM: AML (30%), MM for auto-HCT, AML for allo-HCT.	Acute respiratory failure (hypoxemia, pneumonia, ARDS, sepsis).	Mixed: active/recent hematologic malignancies, post-auto/allo HCT.	ICU mortality: 35% overall (33% HM no HCT, 39% auto-HCT, 40% allo-HCT). Hospital mortality: 45% overall (44% HM, 46% auto, 51% allo). 62% required IMV.	90-day mortality ~50% across groups. No structured QoL or return-to-treatment data.	Independent mortality predictors: ECOG ≥2 (OR 2.25 for 2; OR 5.33 for 3), vasopressor use (OR 2.76), renal replacement therapy (OR 3.07), older age, bronchoalveolar lavage (OR 1.35). HCT type (auto/allo) not associated with increased mortality vs non-HCT HM.	Historically, allo-HCT considered "futile" in ARF — but this study shows comparable survival to non-transplant HM. Suggests ICU admission should not be denied solely on HCT status.

41	Nadkarni AR, et al, 2021 [59]	Global (28 studies, 1,276 ICU patients) Systematic review + meta-analysis	Adult cancer patients (solid and hematologic). Mostly hospitalized during first wave of COVID-19 pandemic (2019–2020).	Severe COVID-19 requiring ICU (respiratory failure, ARDS, sepsis, shock).	Mixed; many on active treatment, though heterogeneity high.	Pooled ICU mortality 60.2% (95% CI 53.6–66.7). Pooled mortality in severe COVID (not ICU-admitted) 58.4%. Mechanical ventilation mortality ~49.4%.	Sparse follow-up beyond discharge; no robust data on resumption of therapy.	Hematologic malignancies had worse outcomes (up to 78% mortality). Cancer patients had ~2-fold higher ICU death risk than non-cancer ICU COVID patients (OR 1.92). Recent anticancer therapy and comorbidities did not significantly change outcomes.	ICU mortality for cancer + COVID-19 was high but not prohibitive. Authors conclude ICU admission should not be categorically denied to cancer patients with COVID-19.
42	Ñamendys-Silva SA, et al., 2017 [20]	Mexico Prospective observational (single center, 2008–2015)	60 testicular cancer patients; mean age 28.3±8.2 yrs; 92% non-seminomatous germ cell tumors; 87% stage III; 82% poor prognosis (IGCCCG).	Septic shock (28%), postoperative care (28%), acute respiratory failure (22%).	98% post-orchiectomy; 87% prior chemotherapy; 23% radiotherapy; 31% second-line therapy.	ICU mortality 38.3%, hospital 45%, 6-month 63.3%.	6-month survival 37%. No QoL data.	Independent predictors of death: ≥2 organ failures in first 24h (HR 3.86), high WBC (HR 1.06), ionized calcium (HR 1.23).	Even though testicular cancer is highly curable, advanced-stage disease + multi-organ failure drastically worsen prognosis. Authors advise early ICU transfer, not delayed until multi-organ failure.

43	Nassar AP Jr., et al., 2020 [28]	Brazil (94 ICUs across 55 hospitals) Retrospective multicenter cohort	4,604 patients ≥80 years with active cancer; mean age 85.6; 91% solid (19.9% metastatic), 9% hematologic.	Unplanned medical or emergency surgical ICU admission.	Active cancer (metastatic or hematologic associated with higher mortality).	ICU mortality 21.3%, hospital mortality 39.2%. Mortality higher in medical (41%) vs emergency surgical (33%) admissions.	No long-term data available.	Independent predictors of hospital mortality: metastatic solid cancer (OR 2.46), hematologic malignancy (OR 2.32), ECOG 2–4 (OR 1.59), vasopressor use (OR 4.74), mechanical ventilation (OR 1.54), renal replacement therapy (OR 1.81).	Demonstrates that very elderly (≥80 y) cancer patients can benefit from ICU admission when functionally preserved and without multi-organ failure. Mortality similar to elderly without cancer in other cohorts. Type of cancer and acute organ dysfunction drive prognosis more than age.

44	Nassar Junior AP, et al, 2023 [17]	Brazil (AC Camargo Cancer Center, São Paulo) Retrospective observational cohort	714 adults with active solid or hematologic malignancy admitted to ICU (2017–2019). 140 (19.6%) colonized with carbapenem-resistant Gram-negative bacteria (CR-GrN) and/or vancomycin-resistant enterococci (VRE). Median age 64.	Unplanned ICU admission in most cases (71.8%). Reasons: medical (sepsis, ARF, shock) or postoperative emergency care.	80% solid, 19% hematologic cancers; 44% metastatic disease. All active cancer under treatment or recently treated.	In-hospital mortality: colonized 44.3% vs non-colonized 33.4% ($p<0.01$); adjusted OR = 1.03 (95% CI 0.77–1.99). Not independently associated with mortality after adjustment.	1-year survival: adjusted HR = 1.10 (95% CI 0.87–1.40); no significant association. Conversion from colonization to infection occurred in 20.7% (mostly CR-GrN).	Poor performance status (ECOG ≥ 2), higher SAPS 3, hematologic malignancy, and unplanned admission predicted mortality. Colonization not an independent prognostic factor.	First ICU-based cancer study linking MDRO colonization to short- and long-term outcomes. Concluded that colonization reflects poor baseline health but does not by itself worsen ICU or 1-year survival. Suggests that screening helps risk stratification but should not justify therapeutic limitation.
45	Nazer L, et al., 2022 [35]	Global (10 studies, multicontinental) Systematic review + meta-analysis (10 observational studies, n=6,605 cancer patients with sepsis)	Hematological (n≈2,814) and solid tumors (n≈3,731). Mean age 51–65, ~55% male.	Sepsis, severe sepsis, septic shock.	Mixed populations: included solid tumors, hematologic malignancies, some with neutropenia, thrombocytopenia, recent chemotherapy. Excluded HSCT and post-surgical dominant cohorts.	Pooled ICU mortality 48% (95% CI 43–53%), hospital mortality 62% (95% CI 58–67%), 28/30-day mortality 50% (95% CI 38–62%). ICU LOS weighted mean 7 days.	Sparse data on return to cancer therapy or long-term survival beyond 6 months. Most follow-up ≤90 days.	Higher mortality associated with septic shock, mechanical ventilation, renal replacement therapy, and hematologic malignancies. Outcomes worse when using SEPSIS-3 definitions (ICU mortality ~68%).	First pooled analysis of ICU sepsis in cancer. Concluded ~2/3 die in hospital, far higher than sepsis in non-cancer ICU patients (~30–40%). Strong signal of poor prognosis for cancer + sepsis, especially with organ support needs.

46	Otten M, et al., 2025 [23]	Netherlands (Amsterdam UMC & UMC Utrecht) Retrospective multicenter matched cohort (2011–2023)	555 adults with hematological malignancies (36% myeloid leukemia, 18% lymphoma, 13% myeloma) matched 1:4 to 2220 controls by APACHE IV and year. Mean age 58.3 ± 13.9.	Respiratory (31%), cardiovascular (32%), hematologic (23%), metabolic (3%), neurologic (4%).	Mixed; 29% allogeneic SCT, 6% autologous SCT, majority with active disease.	ICU mortality 50.3%; 90-day 50%; 1-year 63.8% (vs. 43.7% in controls).	1-year survival 36%; mortality rises with organ support intensity: RRT ≥7 days → 79%, ≥3 organs supported → 84–94%.	↑ Mortality: ≥3 organ supports, prolonged RRT or transfusion, hematologic malignancy itself. Stable mortality after prolonged IMV or vasopressors → not futility per se.	Landmark analysis shows duration of RRT and transfusion, not prolonged ventilation or vasopressors, drives 1-year mortality. Challenges assumption that long ICU stay = futility. Provides objective “time-limited trial” data for hematologic malignancy ICU care.

47	Pohlen M, et al., 2016 [29]	Germany (Münster, Munich, Cologne, Augsburg) Retrospective multicenter cohort with validation cohort	451 adult AML patients (training n=187, validation n=264); median age 59 years. 73% de novo AML, 27% secondary AML.	Severe infection (≈50%), respiratory failure, shock, or multiorgan failure requiring ICU.	Mixed: newly diagnosed, in remission, or relapsed/refractory ; some post–allo-SCT.	ICU mortality 58% (training) vs 36% (validation); overall hospital mortality ≈50%.	Among ICU survivors, 1-year survival 69–100% depending on risk group; 3-year survival after ICU discharge 64%.	ICU mortality predictors: PaO_2 <72 mmHg, active AML (non-remission), severe infection at admission, need for hemodialysis or mechanical ventilation. Post-ICU survival predictors: refractory/relapsed disease, prior allo-SCT, low urine output, GCS <8, Hct <25%.	Developed and validated the "AML in ICU" score, outperforming SAPS II, LOD, and SOFA (AUC 0.91 vs 0.71). Demonstrated that ICU admission can be beneficial in selected AML patients—especially those without multiorgan failure and not post-transplant.
48	Praça APA, et al, 2024 [18]	Brazil (A.C. Camargo Cancer Center, São Paulo) Prospective single-center observational cohort (Sept 2019–June 2021, 18-month follow-up)	3348 adults with newly diagnosed solid or hematologic malignancies (20 cancer types); median age 59; 9.3% had early unplanned ICU admission (n=312).	Sepsis (24.4%), respiratory failure (22.1%), altered consciousness (13.8%), urgent surgery (10.9%).	52% had chemotherapy, 46% surgery, 15% radiotherapy before ICU; 10% no treatment yet.	ICU mortality 8.3% (with forgoing LST), hospital mortality ~9%, but the focus was on survivors discharged home.	18-month survival: 87.0% (ICU) vs 93.9% (no ICU), *p*=0.01; early ICU admission independently decreased survival (adjusted HR 1.84, 95% CI 1.29–2.64).	↑ Risk for early ICU: older age (OR 1.01/yr), comorbidities (CCI per point OR 1.33), ECOG 3–4 (OR 3.7), deprivation (OR 3.3), metastatic tumor (OR 3.1), high-grade hematologic malignancy (OR 6.9).	Shows that even ICU survivors with new cancer diagnoses have reduced long-term survival, possibly due to treatment delays, socioeconomic decline, and post-ICU syndrome. Highlights modifiable factors (early intervention, continuity of cancer care, social support).

49	Provencio M, et al., 2021 [30]	Spain (65 hospitals, GRAVID registry) Prospective multicenter observational cohort	447 lung cancer patients with PCR-confirmed COVID-19. Mean age 67, 74% men, 86% smokers. NSCLC 84.5% (adenocarcinoma 51%), SCLC 15.5%. 79% with stage III–IV disease.	COVID-19 infection with pneumonia /respiratory failure. 78% hospitalized; 2% (n=9) admitted to ICU.	60% on active anticancer therapy, mostly first-line chemotherapy.	Overall mortality 32.7% (146/447). ICU mortality not specified, but ICU admission rare (2%). Hospitalization required in >75%.	No long-term survival data. Follow-up focused on hospitalization/acute outcomes.	Poor prognosis linked to advanced disease (stage III–IV), poor performance status, older age, and comorbidities. Active anticancer treatment not significantly associated with higher mortality.	Large prospective registry during COVID-19 pandemic. Highlights rare ICU admissions in lung cancer patients (2%), despite high hospitalization and mortality. Suggests ICU often withheld in this population, raising triage/ethics questions.
50	Puxty K, et al, 2015 [6]	United Kingdom (West of Scotland) Population-based retrospective cohort (linked cancer registry + ICU database, 2000–2011)	118,541 adults with solid (non-hematologic) cancers; median age 69; 52% women.	Any ICU admission within 2 years of cancer diagnosis, for elective, emergency, surgical, or medical causes.	Mixed; most within 3 months of diagnosis; both curative and palliative phases included.	ICU mortality 14.1%; hospital mortality 24.6%. Mortality highest in emergency medical admissions (ICU 41.7%; hospital 60.1%) and lowest in elective surgical (ICU 0.6%; hospital 4.1%).	Not assessed (registry linkage to 2-year window).	Higher mortality: emergency medical admission, organ support, deprivation quintile 1 (most deprived). Lower mortality: elective surgical, no organ support.	5.2% of solid tumor patients required ICU within 2 years. ICU use highest in colorectal and small intestine cancers, lowest in breast and melanoma. 70% admitted within 3 months of diagnosis. Critical illness may meaningfully affect early cancer survival.

51	Puxty K, et al, 2020 [40]	United Kingdom (West of Scotland) Population-based retrospective cohort (linked cancer registry + ICU database)	26,731 adults with lung cancer (398 admitted to ICU; 1.5% of total). Mean age 67 years; 62% male; 92% NSCLC, 8% SCLC.	Respiratory conditions (27.4%), direct malignancy-related complications (20.3%), post-surgical (15.3%), cardiac (10.8%), other (26%).	Mixed; 35% curative intent (mostly surgical), 46% palliative. 15% ICU admissions directly post-surgical.	ICU mortality 41.5%; hospital 58%; six-month 68.8%.	6-month survival 31.2%. No data on return to therapy or QoL.	ICU admission predictors: surgical treatment (OR 7.23), age <75, male sex. Lower odds with: radiotherapy (OR 0.54), chemotherapy (OR 0.52), age >75.	Only 1.5% of lung cancer patients admitted to ICU. Majority admitted early after diagnosis (median 52 days). Younger, curatively treated, male patients more often admitted. ICU use reflects selection bias and postoperative cases rather than true critical illness burden.
52	Puxty K, et al., 2018 [41]	Scotland, UK (16 ICUs, population registry linked to cancer data) Retrospective cohort (2000–2011, n=25,017 surgical ICU patients)	5,462 surgical ICU patients with solid cancers (21.8% of all admissions). Median age 68 vs 62 in non-cancer patients.	Postoperative ICU admission (elective or emergency). Sepsis, GI/liver disease, surgical complications, hemorrhage common.	Cancer diagnosis within 2 years prior to ICU. Mixture of elective and emergency surgical patients.	ICU mortality: 12.2% (vs 16.8% in non-cancer). Hospital mortality: 22.9% (vs 28.1%). Cancer not a major independent predictor after adjustment (OR 1.09).	6-month mortality higher in cancer (31.3% vs 28.2%). At 4 years: 60.9% vs 39.7% mortality. Survival disadvantage emerges after acute recovery.	Mortality strongly linked to: APACHE II ≥20, age ≥65, emergency hospitalization. Admission directly from operating room protective. Tumor type mattered: better outcomes in thyroid, head/neck, kidney; worse in pancreas, lung, liver.	Key finding: short-term ICU outcomes are favorable for surgical cancer patients, but long-term prognosis reflects tumor biology. Supports ICU admission when surgical intent is curative/palliative-beneficial.

53	Ravetti CG, et al., 2020 [33]	Brazil (Hospital das Clínicas, Federal Univ. of Minas Gerais) Prospective, observational pilot study (2017–2018)	26 adults with hematologic malignancies (AML 38%, lymphoma 27%, MM 11%). Median age 50, 54% male.	Acute respiratory dysfunctio n (febrile neutropeni a, nosocomia l pneumonia , CHF, others).	All had oncohematologic malignancy; excluded allogeneic HSCT. 50% required vasopressors.	ICU mortality 42% (11/26). Mortality differed by ventilation: 0% with O_2 or successful NIMV, 57% in NIMV failure, 100% in IMV group.	No follow-up beyond hospitalization. Median ICU stay 5.5 days.	Higher LUS at admission predicted non-survival (13 vs 9, p=0.047). NIV success associated with survival; NIMV failure/intubation with death. SOFA also higher in non-survivors.	LUS may be a helpful bedside prognostic tool in hematologic patients with ARD. Confirms high mortality with IMV, but possible benefit when NIMV succeeds.
54	Saillard C, et al, 2020 [42]	France (Institut Paoli-Calmettes, Marseille) Retrospective observational (2010–2017)	127 cancer patients with ARF + cardiac dysfunction. Median age 66, 60 hematologic malignancies, 48 solid tumors, 27% allogeneic HSCT, 7% autologous HSCT. 63% with progressive disease.	Acute respiratory failure with cardiac dysfunctio n (LVD 91%, RVD 24%, sepsis 82%, infectious pneumonia 46%).	Mix of hematologic and solid tumors, many with progressive disease; ~48% had received cardiotoxic chemo in last 3 months.	ICU mortality 29%, hospital mortality 57%. NIV group: ICU mortality 16% vs 42% in non-NIV strategies (p=0.001). 48% ultimately required intubation.	No long-term follow-up beyond hospital discharge.	Independent mortality predictors: SAPS II score (OR 1.07/point), invasive fungal infection (OR 7.65), ventilation strategy (HFNO alone OR 19.56, standard O_2 alone OR 10.72, both vs NIV). NIV protective in multivariate and propensity score analysis (ICU mortality 10% vs 50%).	Demonstrates NIV as beneficial first-line strategy in cancer patients with ARF + cardiac dysfunction. Hospital mortality still high (57%), but ICU survival improved with early NIV.

55	Scarfò L, Chatzikonstantinou T, Rigolin GM, et al., 2020 [63]	Multinational (mainly Italy, Spain; 118 centers in 30+ countries) Retrospective multicenter observational study (ERIC/CLL Campus)	190 patients with chronic lymphocytic leukemia (CLL) and confirmed COVID-19 (median age 72; 67% male; 76% with comorbidities).	COVID-19 pneumonia; 88.9% hospitalized; 20% ICU (39/190).	34% on active CLL treatment (mainly BTK inhibitors, venetoclax, CIT); 26% recently treated; 38% untreated.	Overall mortality 30%; ICU mortality ≈70% (16/23 ICU deaths). Severe COVID-19 in 79% (need O_2 or ICU); 36% mortality in severe cases.	Median follow-up 23 days; 69% 30-day survival; recovery in 51%.	Severe disease linked to age ≥65 (OR 3.72); untreated or recently off therapy associated with worse severity; ibrutinib users had lower hospitalization risk (OR 0.44); age and comorbidities did not predict mortality once severe disease developed.	Early global snapshot of COVID-19 in CLL. Shows high ICU mortality but possible protective effect of BTK inhibitors. CLL-related immune dysfunction may drive poor outcomes. Suggests selective ICU benefit for patients on active targeted therapy.
56	Shaker EH, et al, 2025 [53]	Egypt (National Cancer Institute, Cairo University) Prospective, double-blind, randomized controlled trial	90 cancer patients with septic shock, aged 18–65. Breast, pancreatic, and colorectal cancers represented. All mechanically ventilated and requiring vasopressors.	Septic shock (per Sepsis-3), all mechanically ventilated. Infection sources: pneumonia, intra-abdominal, urinary tract.	Mixed cancer types, all advanced/active. Oncologic treatment details not stratified, but all under active care at NCI.	ICU mortality: Placebo 46.7%, MB 1 mg/kg 30%, MB 4 mg/kg 20% (p=0.083 overall; HR for high-dose MB 0.29, 95% CI 0.09–0.90). Vasopressor discontinuation faster in MB groups (70–74h vs 94h placebo).	28-day follow-up: vasopressor-free days increased with MB; trend toward survival benefit with 4 mg/kg MB. No longer-term outcomes reported.	Protective: high-dose MB (HR 0.29). Not significant but trend in low-dose MB. Other prognosticators (SOFA, age, comorbidities) not significantly different between groups at baseline.	First RCT in oncology ICU sepsis testing MB. Suggests MB is safe and may improve hemodynamics and short-term survival in septic shock. Novel therapeutic option in high-risk cancer population.

57	Shen J, et al., 2025 [69]	China (37 cancer-specialty hospitals) Multicenter longitudinal observational study	269 adults with primary lung cancer admitted to ICU (median age 65; 71% male).	Sepsis (73%), respiratory failure (52%), ARDS (21%), shock (27%), AKI (13%).	31% on active systemic therapy (chemo 30%, immuno 14%, targeted 20%). 39% post-surgical.	ICU mortality 11.9%; in-hospital 15.2%; 90-day 45.4%.	90-day survival 54.6%. No QoL or therapy resumption data.	Independent predictors of 90-day mortality: ↑ age (HR 0.96), ↓ BMI (HR 0.92), sepsis (HR 2.6), respiratory failure (HR 2.2), AKI grade II–III (HR 2.8 & 2.5), no prior surgery, unplanned ICU admission.	Despite low AKI incidence (12.6%), it was the strongest independent predictor of ICU, hospital, and 90-day mortality. Anti-infective regimen (carbapenems, triazoles) associated
58	Shrime MG, et al., 2016 [4]	USA (Boston; validated in Riyadh, Saudi Arabia) Decision-analytic microsimulation model using MIMIC-II ICU database (n=920 derivation; 3 external validation cohorts)	Critically ill patients with poor-prognosis cancers (solid tumors 70%; hematologic malignancies 30%). Median age 64.	Heterogeneous ICU admissions for respiratory failure, shock, or postoperative care.	Mixed: metastatic, hematologic, and poor-prognosis primary cancers (e.g., GBM, pancreas, lung, melanoma).	Overall 30-day mortality 31.1%; ICU mortality 21%. For solid tumors: 30-day survival 37%, falling to 1.6% with SOFA ≥15.	Mean survival duration modeled: similar between 8–12-day ICU trials and time-unlimited care for moderate illness; no survival gain beyond 4 days in poor-prognosis solid tumors.	Admission SOFA score was the main determinant of survival benefit. For solid tumors, optimal trial length = 1–4 days; for hematologic malignancies or lower SOFA, 8–12 days.	Validated model across 3 centers showed that short, time-limited ICU trials (≤4 days) may be adequate for poor-prognosis solid tumors, while longer trials (up to 2 weeks) are justified for hematologic or less severely ill patients. Provides empirical definition for “futility thresholds” in oncology ICU care.

59	Šimkovič M, et al., 2023 [64]	Czech Republic (9 hematology centers, Czech CLL Study Group) Multicenter retrospective cohort	341 adults with chronic lymphocytic leukemia (CLL) and confirmed COVID-19 (median age 69; 70% male).	COVID-19 pneumonia; 60% hospitalized, 21% ICU, 12% mechanical ventilation.	63% previously treated for CLL; 28% receiving therapy at COVID-19 diagnosis (48% BTKi, 26% chemoimmunotherapy, 18% Bcl-2 inhibitor).	Overall CFR 28%; ICU CFR 73%. Hospitalized CFR 45%; non-hospitalized CFR 1.6%.	Median follow-up 15.6 weeks; no long-term post-COVID data.	Independent predictors of death: prior or active CLL therapy, use of steroids for COVID-19, age >72, comorbidities, male sex. Chemoimmunotherapy and BTKi had similar risks.	Confirms very high mortality in ICU CLL patients (≈70%). However, patients on targeted therapy who continued BTKi or venetoclax did *not* die — suggesting selective benefit. Mortality decreased from 32% (2020) to 24% (2021). Vaccination effect minimal (only 4 patients vaccinated pre-infection).
60	Singh I, et al., 2024 [37]	USA (Memorial Sloan Kettering Cancer Center, NY) Retrospective single-center observational study (2014–2020)	124 patients with stage IV PDAC and ≥1 documented episode of acute cholangitis; median age 64; 54.8% male.	12 patients (9.7%) admitted to ICU for sepsis or organ failure due to cholangitis.	62.1% on systemic therapy (FOLFIRINOX or gemcitabine/nab-paclitaxel). 91.9% had prior biliary intervention.	4% in-hospital mortality; 30-day survival 86.2%; 6-month 37%; 12-month 18.9%.	Median OS 4.1 months after first AC episode (95% CI 4.0–5.5). Median OS from PDAC diagnosis ~8.3 months. 34% had recurrent cholangitis within median 2.3 months.	Worse outcomes with: tumor in body/tail (HR 2.29), liver + extrahepatic metastases (HR 1.96), grade 3 cholangitis (HR 2.26). ICU admission, sepsis, timing of biliary drainage, or chemo regimen did not predict survival.	AC is a major cause of morbidity and short-term mortality in advanced PDAC. ICU-level complications often mark transition to preterminal phase. Calls for prevention (better stent durability, earlier drainage).

61	Smith M, et al., 2020 [65]	USA (New York City, 5 academic centers) Multicenter retrospective cohort	86 patients with gynecologic cancer (ovarian ≈ 50%, endometrial ≈ 30%, cervical ≈ 20%); median age 68.5 (IQR 59–75)	COVID-19 pneumonia; severe infection defined as ICU admission, ventilation, or death	50 (58%) active cancer, 36 (42%) in remission	ICU admission 33.7% (29/86); mortality 29.1% (25/86); all deaths COVID-related	Short-term only; no follow-up beyond hospitalization	Elevated ferritin > 1000 ng/mL, procalcitonin > 0.5 ng/mL, CRP > 100 mg/L, plus ↑ WBC, lactate, creatinine → predictors of severe infection. D-dimer not predictive.	Identified inflammatory biomarke
62	Storck A, et al., 2025 [36]	Germany & Austria (9 centers; iCHOP registry) Multicenter retrospective registry study (2014–2021)	1,762 adult cancer patients; 106 (6%) received targeted therapy (TT). Median age 62 (IQR 53–70). 57% hematologic malignancies (notably NHL 42%), 43% solid tumors.	Respiratory failure (34%), shock (16%), infection (15%), neurologic (11%), other (24%).	Mixed: 35% treatment-naïve, 20% progressive disease, 11% complete remission. 23% prior HSCT (mostly autologous). 54% received TT + chemo; 45% TT alone.	ICU mortality 39%; hospital mortality 48%; median OS 44 days. TT cohort ICU survival 61%, hospital survival 52% (comparable to non-TT cohort).	No QoL data. Median hospital stay longer in TT cohort (40 vs 26 days). Trend toward improved survival for TT recipients (esp. TT+chemotherapy).	Independent predictors of mortality: progressive disease (HR 1.43), SOFA score (HR 1.12 per point), mechanical ventilation, renal replacement therapy (HR 1.22), advanced directives (HR 1.85).	First comprehensive ICU study of targeted therapy. Demonstrates feasibility and potential benefit of TT even in critically ill cancer patients—outcomes similar to or slightly better than non-TT despite higher risk. Highlights the impact of disease status at ICU admission.

63	Tanguy-Melac A, et al., 2019 [7]	France (nationwide, CNAM/SNDS database) Retrospective national observational study	15,361 individuals with colorectal cancer (CRC) who died in 2015; mean age 75 years (SD 12.5); 43% ≥80 years; 44% female. One-third had another active cancer.	17% admitted to ICU during last month of life for acute organ failure or sepsis (administrative data). 39% had ≥1 ER visit.	All had CRC; 60% received hospital palliative care (HPC) in the last year; 15% had chemotherapy within last month of life (<60 years: 27%).	17% ICU admission during last month; 83% hospitalized in last 30 days; 66% died in short-stay hospital (SSH). Mean hospital stay: 71 days/year.	Not reported (end-of-life population).	Aggressive end-of-life care (ICU, chemotherapy) associated with younger age, fewer comorbidities, absence of palliative care; HPC associated with reduced ICU and chemo use.	Demonstrates high-intensity medical care at end of life in French CRC patients. Despite national palliative programs, hospital-centered deaths (66%) and late transition to palliative care persisted. Suggests need for earlier HPC integration and ICU triage discussions.
64	Teng X, et al, 2023 [73]	China (review of global studies, 1998–2022) Narrative review (24 studies: 10 retrospective, 1 prospective, 13 case reports)	Solid tumors (lung, esophageal, mediastinal, tracheal, etc.); hematologic malignancies (leukemia, lymphoma, multiple myeloma); post-HSCT patients. N varied across included studies.	Severe acute respiratory failure (VV-ECMO), cardiogenic shock (VA-ECMO), perioperative cardiopulmonary support, chemotherapy bridging.	Mixed: newly diagnosed, relapsed, HSCT, chemotherapy-sensitive tumors, perioperative patients.	Reported ICU/hospital survival highly variable: ~20–32% survival in hematologic malignancy cohorts; up to 30–50% in selected solid tumor cases (e.g., thoracic surgery). HSCT patients with ARDS had extremely poor outcomes (0–19% survival).	Some survivors achieved long-term remission (e.g., 36-month survival in select lymphoma cases; Karnofsky 100 after chemo + ECMO). Case reports show feasibility of bridging to curative surgery or chemotherapy.	Negative: progressive/refractory malignancy, HSCT within 240 days, severe neutropenia, thrombocytopenia. Positive: chemo-sensitive disease, high functional status, perioperative planned use, multidisciplinary selection.	Cancer is not an absolute contraindication for ECMO. May be beneficial in selected patients (solid tumors with surgical/chemo options, lymphoma in remission) but generally poor for acute leukemia and early post-HSCT ARDS. Calls for multicenter prospective studies.

65	Van Matre ET, et al., 2018 [11]	USA (566 hospitals, Premier Database) Retrospective, propensity-matched comparative effectiveness cohort	103,798 critically ill adults with solid tumors (non-metastatic & metastatic) or lymphoma (age >18). After matching: 63,561 (42,343 LMWH vs. 21,218 UFH).	All ICU admissions; specific diagnoses included respiratory failure (14%), sepsis (6%), renal failure (12%), cardiac failure (15%).	Mixed; most admitted during treatment (within 1 year of diagnosis).	In-hospital mortality 7.9% (LMWH) vs. 7.6% (UFH) — no difference. VTE 5.3% vs. 5.5%; PE 0.7% vs. 1.0% (↓ with LMWH). Significant bleeding 13.3% vs. 14.8% (↓ with LMWH). HIT 0.06% vs. 0.19% (↓ with LMWH).	No follow-up beyond hospitalization.	LMWH → ↓ PE (OR 0.7, p<0.001), ↓ bleeding (p<0.001), ↓ HIT (p<0.001). No mortality difference. LMWH improved ICU LOS (–0.5 days) and total hospital LOS (–0.2 days).	Largest multicenter ICU cohort comparing anticoagulants in oncology. Shows LMWH safer and equally effective, especially in non-metastatic solid tumors. Reinforces LMWH as preferred ICU prophylaxis.
66	Wei M, et al., 2023 [48]	China (33 ICUs in 26 provinces) Multicenter retrospective cohort (real-world, 2-month data collection)	1488 cancer patients; 922 with unplanned ICU admissions. Mean age 62.2 years, 62% male. Main cancers: esophageal (36%), lung (22%), abdominal (14%), gynecological (9%).	Sepsis (81%), respiratory failure (51%), shock (36%), AKI (17%), bone marrow suppression (18%).	33% received chemotherapy within 3 months, 8.5% radiotherapy, others on targeted/immunotherapy. 27% received antitumor therapy after ICU transfer.	ICU mortality 9.1%; hospital mortality 11.1%. Median ICU stay 5 days, hospital stay 19 days.	90-day mortality 40.8% (n=376). 59% of unplanned ICU patients survived 90 days. Tumor progression caused 58% of deaths. ~27% resumed cancer treatment after ICU discharge.	Protective: higher BMI, history of hypertension. Risk factors: recent antitumor therapy (<3 months), transfer from ward/ED/external hospital, high APACHE II, shock, respiratory failure, invasive ventilation, AKI, renal replacement therapy.	First large Chinese study of unplanned ICU admissions in oncology. Developed a risk prediction model (nomogram) with 8 variables, C-index 0.77. Highlights importance of early recognition and timely ICU admission.

67	Xia R, et al, 2016 [49]	China (Tianjin Medical Univ. Cancer Institute) Retrospective observational, single center (2012–2015)	141 advanced solid tumor patients (from 813 ICU admissions). Median age 63, 62% male. Tumors: stomach 23%, pancreas 13%, lung 11%, rectal/colon 14%. All metastatic.	Respiratory failure (38%), sepsis/septic shock (28%), renal failure, cardiac failure.	All advanced, metastatic. 51% prior chemotherapy, 12% radiotherapy, 4% biological therapy. 87% prior surgery.	ICU mortality 14.9%; in-hospital 29.8%. With interventions: vasopressors >24h mortality 25%, MV >24h 25.9%, RRT >24h 40%.	Mean OS 28.6 months; 21% resumed anticancer treatment after ICU. ICU survivors had median OS ~30 months vs ~17 months in non-survivors.	Only SOFA score at day 7 predicted ICU mortality (OR 1.61). APACHE II day 1 predicted long-term OS. Hypertension, lung cancer, and need for MV associated with worse short-term outcomes. Early intervention (shorter time to ICU) improved survival.	Authors advocate an “ICU trial” for advanced solid tumor patients, with full support for 6–7 days, then reassessment. Suggest broadening admission criteria — cancer stage/type not independently predictive; SOFA at day 7 is the critical decision point.

68	Xia R., Wang D., 2019 [50]	China (Tianjin Medical University Cancer Institute & Hospital) Retrospective single-center observational cohort	229 postoperative gastrointestinal cancer patients (gastric 49.8%, colon 20.1%, esophageal 18.3%); median age 63 years (IQR 54–74); all non-neutropenic.	Postoperative critical care after major abdominal oncologic surgery (e.g., gastrectomy, colectomy, esophagectomy).	Active solid tumors post curative surgery; no hematologic malignancy.	ICU mortality 5.7%, in-hospital mortality 7.4%. Invasive candidiasis (IC) occurred in 8.3% (19/229), candidemia in 2.6%. Mortality for IC 15.8%, for candidemia 33.3%.	No long-term follow-up reported.	Independent predictors: Corrected Candida Colonization Index (CCI) ≥0.4 (OR 66.4, p=0.000) for IC; number of organ failures for candidemia (OR 0.184, p=0.026).	CCI ≥0.4 best predictor for intra-abdominal candidiasis; BDG ≥80 pg/mL had poor sensitivity (25%) but high negative predictive value (95.7%) for candidemia. Multifocal Candida colonization highest in cardiac/gastric surgery. Suggests targeted, not empirical, antifungal use in oncology ICUs.
69	Xie Z, et al., 2021 [13]	USA (Mayo Clinic, 3 major + 5 satellite campuses) Prospective observational, quality improvement (Mar–Jul 2020)	224 cancer patients with COVID-19. Median age 54, 42% female. 175 solid tumors, 49 hematologic. 33% on active cancer-directed therapy (CDT).	COVID-19 infection. ICU admission in 10 patients (4.5%).	Mixed: chemo (16%), immunotherapy (5%), targeted (14%), endocrine (11%), CAR-T/HSCT small subset.	COVID-19 hospitalization 24%; ICU admission 4.5% (10/224). Mortality 4% (9/224). IMV required in 8 (3.6%), NIPPV in 7 (3.2%).	No structured long-term follow-up; median hospitalization 6 days. Some resumed therapy after clearance.	Independent predictors of hospitalization: age ≥60 (OR 4.4), lymphopenia <1.4 × 10^9/L (OR 9.2). Active CDT not associated with worse outcomes.	Showed that routine screening identified asymptomatic infections but did not reduce ICU or mortality outcomes vs clinical testing. Mortality lower than in other COVID-cancer cohorts (4%).

70	Yang K, et al., 2020 [45]	China (Hubei, 9 hospitals) Retrospective multicenter cohort (Jan–Mar 2020)	205 cancer patients with COVID-19. Median age 63 (range 14–96). 183 solid tumors (breast 20%, colorectal 14%, lung 12%) and 22 hematologic (11%).	COVID-19 pneumonia /respiratory failure. 30/205 (15%) required ICU.	54/182 (30%) received anticancer therapy within 4 weeks of COVID onset. Mix of chemo, immunotherapy, targeted therapy.	Overall hospital mortality: 20% (40/205). Hematologic malignancy mortality 41% vs 17% for solid tumors (HR 3.28). ICU mortality higher than non-ICU, but subgroup not numerically detailed.	Median follow-up 68 days. No long-term therapy resumption reported.	Independent predictors of death: chemotherapy within 4 weeks pre-COVID (OR 3.51), male sex (OR 3.86), hematologic malignancy (HR 3.28). Age, stage, smoking not independently significant.	Among largest early cancer + COVID ICU cohorts. Confirms high risk in hematologic cancers and recent chemotherapy. ICU use frequent (15%), but survival possible, especially for solid tumors.
71	Zaki A, et al., 2022 [66]	Pakistan (5 tertiary centers) Multicenter retrospective cohort (Feb–Aug 2020)	107 hematologic malignancy patients, median age ~50. Diagnoses: acute leukemias 29%, NHL 27%, HL 18%, MM 7%, CLL 6%, CML 5%.	COVID-19 infection (PCR confirmed). 25 deaths, of which 14/25 (56%) were admitted to ICU, median stay 11 days.	Majority on active treatment (84% in dead vs 59% in survivors). Common: IV chemo, chemoimmunotherapy, TKIs.	Overall mortality 23.4%. Among hospitalized: ~51% mortality. Among ICU patients: 86% mortality.	Median overall survival 33 days (IQR 15–60). No long-term return-to-treatment reported.	Independent risk factors (Cox regression): age >50, being on IV chemotherapy (HR 1.51), chemoimmunotherapy (HR 1.46), contact/travel exposure (HR 2–3x higher), being COVID-positive at discharge (HR 3.41). Hypertension also overrepresented in non-survivors.	Shows extremely high ICU mortality (86%) in hematologic malignancy patients with COVID-19 in LMIC setting. Active treatment increased risk. Provides important contrast with high-income datasets.

72	Zhang H, et al, 2024 [21]	China (Lianyungang Hospital, Xuzhou Medical University) Prospective observational cohort with PSM	245 CRC surgery patients (Aug 2021–May 2023). Median age 67, 60% male. Excluded distant metastases. 52.6% frail by Fried phenotype.	Postoperative complications — subset required ICU (not all admissions elective).	Elective radical surgery only. Non-metastatic colon or rectal cancer.	ICU admission: 8.2% overall (20/245). 30-day mortality 1.2%, 90-day mortality 1.6%. No significant difference between frail vs non-frail after PSM.	No long-term survival follow-up beyond 90 days.	Preoperative frailty was an independent predictor of intra-abdominal infections (OR 12.0, 95% CI 1.33–108, p=0.027). BMI and comorbidities also influenced infection risk. Frailty tended to increase ICU admission and 90-day mortality, though not statistically significant.	Demonstrates that frailty screening predicts surgical complications in CRC. Short-term survival after ICU was high, but frailty correlated with morbidity (infections, prolonged stay).
73	Zhang L, et al., 2020 [56]	China (Wuhan, 3 hospitals) Retrospective multicenter case series	28 adults with solid tumors and laboratory-confirmed COVID-19. Median age 65 (56–70); 61% male; 36% stage IV. Lung cancer 25%, esophagus 14%, breast 11%.	Severe events defined as ICU admission, mechanical ventilation, or death. 6 (21%) admitted to ICU.	100% had prior anti-tumor therapy; 21% received active treatment within 14 days (chemo = 3, targeted = 2, RT = 1, immunotherapy = 1).	Severe events in 53.6%; ICU 21.4%; death 28.6%. Median time from diagnosis to death = 16 days. ARDS most common complication (29%).	No follow-up beyond hospitalization; 36% discharged; 36% still hospitalized at study close.	Recent anti-tumor treatment (<14 days) → HR 4.08 (95% CI 1.09–15.32, p = 0.037). Patchy CT consolidation → HR 5.44 (95% CI 1.50–19.75, p = 0.010).	COVID-19 caused rapid deterioration and high mortality, especially in those on recent cytotoxic or targeted therapy. Lung cancer patients had earlier dyspnea (median 1 day vs 5 days non-lung cancer). Study urged delay or dose modification of immunosuppressive therapies during infection.
